# Modeling cell line-specific recruitment of signaling proteins to the insulin-like growth factor 1 receptor

**DOI:** 10.1371/journal.pcbi.1006706

**Published:** 2019-01-17

**Authors:** Keesha E. Erickson, Oleksii S. Rukhlenko, Md Shahinuzzaman, Kalina P. Slavkova, Yen Ting Lin, Ryan Suderman, Edward C. Stites, Marian Anghel, Richard G. Posner, Dipak Barua, Boris N. Kholodenko, William S. Hlavacek

**Affiliations:** 1 Theoretical Biology and Biophysics Group, Theoretical Division, Los Alamos National Laboratory, Los Alamos, New Mexico, United States of America; 2 Systems Biology Ireland, University College Dublin, Belfield, Dublin, Ireland; 3 Department of Chemical and Biochemical Engineering, University of Missouri Science and Technology, Rolla, Missouri, United States of America; 4 Center for Nonlinear Studies, Los Alamos National Laboratory, Los Alamos, New Mexico, United States of America; 5 The Salk Institute for Biological Studies, La Jolla, California, United States of America; 6 Information Sciences Group, Computer, Computational and Statistical Sciences Division, Los Alamos National Laboratory, Los Alamos, New Mexico, United States of America; 7 Department of Biological Sciences, Northern Arizona University, Flagstaff, Arizona, United States of America; 8 School of Medicine and Medical Science and Conway Institute of Biomolecular and Biomedical Research, University College Dublin, Belfield, Dublin, Ireland; Northeastern University, UNITED STATES

## Abstract

Receptor tyrosine kinases (RTKs) typically contain multiple autophosphorylation sites in their cytoplasmic domains. Once activated, these autophosphorylation sites can recruit downstream signaling proteins containing Src homology 2 (SH2) and phosphotyrosine-binding (PTB) domains, which recognize phosphotyrosine-containing short linear motifs (SLiMs). These domains and SLiMs have polyspecific or promiscuous binding activities. Thus, multiple signaling proteins may compete for binding to a common SLiM and vice versa. To investigate the effects of competition on RTK signaling, we used a rule-based modeling approach to develop and analyze models for ligand-induced recruitment of SH2/PTB domain-containing proteins to autophosphorylation sites in the insulin-like growth factor 1 (IGF1) receptor (IGF1R). Models were parameterized using published datasets reporting protein copy numbers and site-specific binding affinities. Simulations were facilitated by a novel application of model restructuration, to reduce redundancy in rule-derived equations. We compare predictions obtained via numerical simulation of the model to those obtained through simple prediction methods, such as through an analytical approximation, or ranking by copy number and/or K_D_ value, and find that the simple methods are unable to recapitulate the predictions of numerical simulations. We created 45 cell line-specific models that demonstrate how early events in IGF1R signaling depend on the protein abundance profile of a cell. Simulations, facilitated by model restructuration, identified pairs of IGF1R binding partners that are recruited in anti-correlated and correlated fashions, despite no inclusion of cooperativity in our models. This work shows that the outcome of competition depends on the physicochemical parameters that characterize pairwise interactions, as well as network properties, including network connectivity and the relative abundances of competitors.

## Author summary

Cells rely on networks of interacting biomolecules to sense and respond to environmental perturbations and signals. However, it is unclear how information is processed to generate appropriate and specific responses to signals, especially given that these networks tend to share many components. For example, receptors that detect distinct ligands and regulate distinct cellular activities commonly interact with overlapping sets of downstream signaling proteins. Here, to investigate the downstream signaling of a well-studied receptor tyrosine kinase (RTK), the insulin-like growth factor 1 (IGF1) receptor (IGF1R), we formulated and analyzed 45 cell line-specific mathematical models, which account for recruitment of 18 different binding partners to six sites of receptor autophosphorylation in IGF1R. The models were parameterized using available protein copy number and site-specific affinity measurements, and restructured to allow for network generation. We find that recruitment is influenced by the protein abundance profile of a cell, with different patterns of recruitment in different cell lines. Furthermore, in a given cell line, we find that pairs of IGF1R binding partners may be recruited in a correlated or anti-correlated fashion. We demonstrate that the simulations of the model have greater predictive power than protein copy number and/or binding affinity data, and that even a simple analytical model cannot reproduce the predicted recruitment ranking obtained via simulations. These findings represent testable predictions and indicate that the outputs of IGF1R signaling depend on cell line-specific properties in addition to the properties that are intrinsic to the biomolecules involved.

## Introduction

Cellular regulatory networks encompass conserved patterns of protein-protein interactions, which have been called network motifs [1,2]. One key network motif is the phosphotyrosine-based reader/writer/eraser motif [3,4], which is found in many important human cell signaling systems, including systems downstream of antigen, cytokine, and hormone receptors. The motif consists of 1) a tyrosine residue within a protein, 2) a kinase that phosphorylates the tyrosine (termed a writer), 3) a phosphatase that dephosphorylates the phosphotyrosine product of kinase activity (termed an eraser), and 4) a protein containing a Src homology 2 (SH2) and/or phosphotyrosine binding (PTB) domain (termed a reader). SH2 and PTB domains are conserved modular structural units consisting of approximately 100 amino acids that have the ability to recognize and bind phosphotyrosine-containing short linear motifs (SLiMs) [5,6]. Binding properties of SLiMs are determined by regions generally spanning three to ten amino acids [6]. SH2 and PTB domains have preferences for specific SLiMs, which confers a degree of specificity in phosphotyrosine signaling [7]. However, interactions are characterized by equilibrium dissociation constants (*K*_D_ values) that fall roughly between 1 and 10 μM, a range not thought to be large enough to confer a high degree of specificity [8–11]. Thus, neither SH2/PTB domains nor SH2/PTB domain-binding SLiMs have exclusive and unique binding partners. Rather, instances of these domains and SLiMs each have the potential to interact with multiple binding partners, a property that is sometimes referred to as binding promiscuity [12–14].

The consequences of binding promiscuity are not well understood. It has been suggested that promiscuity may necessitate a strengthening of reader/writer/eraser signaling specificity by factors such as compartmentalization or secondary protein-protein interactions beyond the characteristic phosphotyrosine-SH2/PTB domain interaction [15]. However, the specificity requirements of cellular information processing are unclear. It may be that limited specificity is sufficient for the function of the reader/writer/eraser motif, and it may even be that numerous interactions are not merely tolerated but somehow important for proper functioning [16]. In any case, a byproduct of binding promiscuity is competition.

Competition arises as a factor that influences reader/writer/eraser motif function because we expect that only a single SH2/PTB domain can physically interact with only one phosphotyrosine at a time. Thus, the recruitment of an SH2/PTB domain to a phosphotyrosine may be significantly influenced by the presence of other interaction partners [12]. Which SH2/PTB domain containing proteins are bound first may even influence the ability of potential competitors to bind, through a protective shielding effect [17]. Importantly, competition is a network property, making mathematical models for network behavior potentially valuable for integrating information about relative abundances of SH2/PTB domains and phosphotyrosines and affinities of pairwise interactions and for reasoning about the effects of competition [18].

Here, we formulated a series of models to investigate signaling by a well-studied receptor tyrosine kinase (RTK), the insulin-like growth factor 1 (IGF1) receptor (IGF1R). In normal cells, IGF1R signaling regulates differentiation, proliferation, motility, and anti-apoptosis; however, IGF1R signaling has also been strongly linked to the progression of cancer [19,20], with mutations affecting the IGF1R or the related insulin receptor (INSR) signaling pathway noted in 43% of stomach cancer tumors [21] and activated IGF1R detected in 50% of breast cancers across multiple subtypes [21,22]. Furthermore, loss of IGF1R signaling in pancreatic β cells has been observed to promote diabetes [23,24]. Models capable of predicting IGF1R signaling pathway behavior could aid in the realization of effective treatment strategies for IGF1R-driven disease.

Mass-action models of cell signaling typically focus on a limited subset of signaling proteins [25,26] or group a set of binding partners together [27] because of the challenge of accounting for a combinatorial number of phosphoforms and protein complexes with traditional modeling approaches [28]. For an IGF1R homodimer with six phosphotyrosines per monomer, if each phosphotyrosine is limited to one interaction, there are three possible states (unphosphorylated/unbound, phosphorylated/unbound, or phosphorylated/bound) for each of the 12 phosphotyrosines in the homodimer. Overall, there are on the order of 10^5^ unique dimer configurations that can be populated. This complexity naturally follows from biological mechanisms and presents a challenge to those who wish to develop and analyze mathematical models to better understand cell signaling.

Rule-based modeling was developed to cope with combinatorial complexity [29]. This approach allows reactions to be defined in terms of rules describing molecular interactions mediated by specific parts of proteins. A list of rules can be used to computationally generate the entire network of molecular states and reactions implied by the interactions represented by the rules. Such a comprehensive specification might be needed to simulate a network through numerical integration of a system of ordinary differential equations (ODEs) [30,31]. However, the combinatorial explosion in number of potentially populated chemical species that occurs as more interactions are considered often leads to models with a very large number of mostly redundant states that cannot be efficiently derived from rules or efficiently simulated with ODE-based approaches. For an example of redundancy, consider a receptor with *n* tyrosine sites that can be either unphosphorylated or phosphorylated. Describing changes to every possible configuration of a receptor would require 2^*n*^ ODEs. However, if the state of one tyrosine residue does not influence the state of others, then the same system of interactions could be fully captured with only 2*n* equations. One way to overcome the combinatorial explosion problem is with network-free simulation algorithms that avoid the explicit specification or derivation of all possible states [32–36]. A second option is model reduction, in which an approximate model is derived by neglecting sparsely populated species [37]. With this approach, a network and equations must be derivable from rules, then the derived network and equations are simplified according to the results of simulation. In this report, we applied a method of restructuring a model formulation to reduce state redundancy, which allows the model to be simulated with network-based algorithms. Strategies similar to the restructuration approaches employed here have been previously described [38–43]. In contrast to model reduction, model restructuration does not entail approximation to arrive at a simpler model form.

We applied a rule-based approach to formulate mathematical models for early events in IGF1R signaling. We modeled IGF1 binding to IGF1R based on work by Kiselyov et al. [44], which we built upon by considering the full-scale interaction network of IGF1, IGF1R, and a set of IGF1R binding partners. We leveraged the availability of datasets characterizing interaction affinities between IGF1R and a subset of the human complement of SH2/PTB domains [45,46]. Importantly, we demonstrate that naive predictors of signaling protein recruitment, including binding affinity, copy number, and simple analytical expressions for equilibrium binding, are unable to recapitulate predictions obtained via simulations. Using cell line-specific measurements of protein copy numbers, we extended the model to make predictions for IGF1R binding partner recruitment across diverse cell lines. Thus, this work considers the effects of competition for phosphotyrosine sites, differences in binding affinity, and the impacts of cell line-specific protein abundance profiles to rank the importance of downstream IGF1R signaling partners.

## Results

### Formulating cell line-specific models of IGF1R signaling

**Fig 1:**
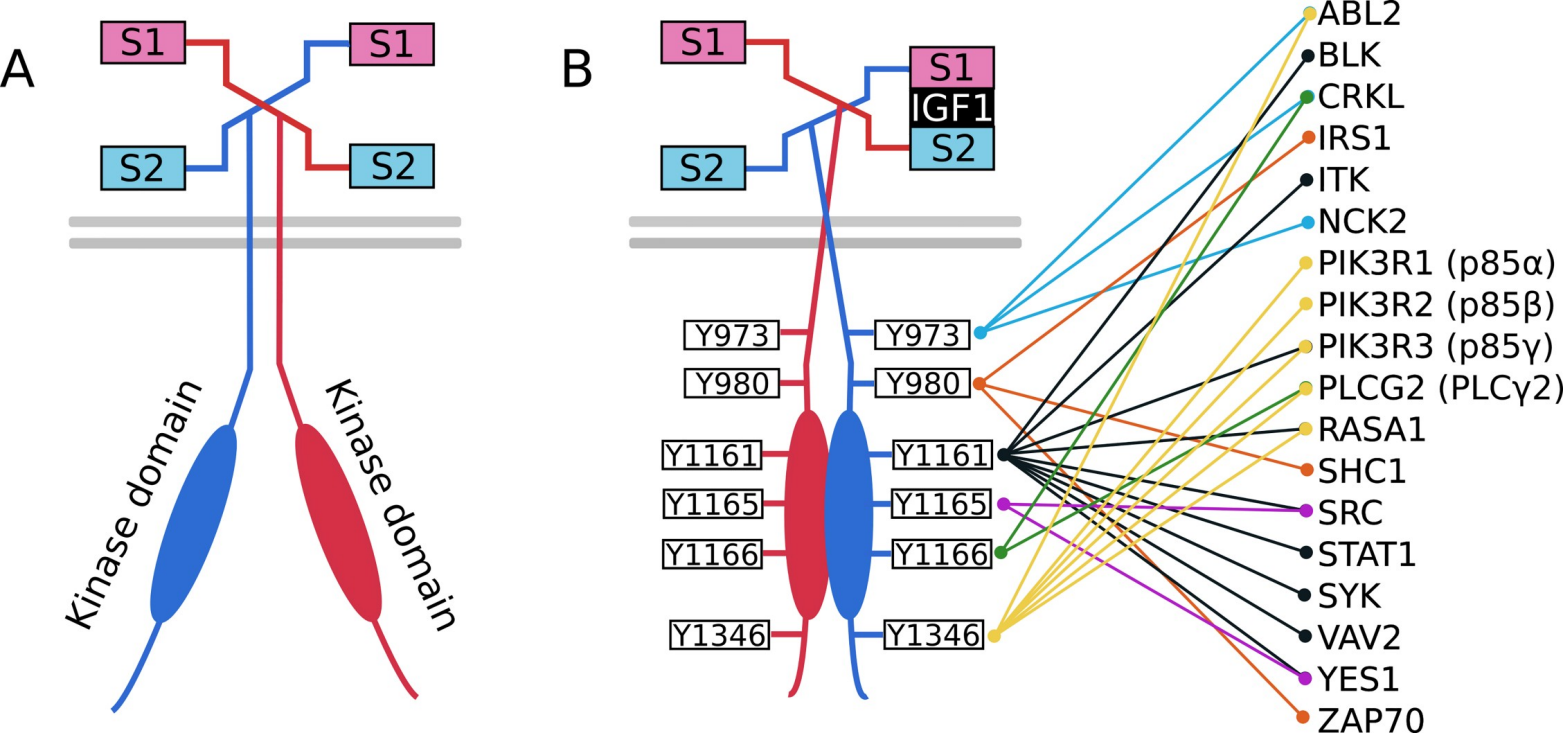
IGF1R phosphotyrosine contact map in HeLa S3 cells. (A) Ligand-free and inactive IGF1R. Prior to crosslinking of dissimilar sites S_1_ and S_2_ of each IGF1R protomer in a dimer, the two kinase domains are not in contact and are thus unable to autophosphorylate receptor tyrosines. (B) IGF1R phosphotyrosine interactions in HeLa S3 cells. Each IGF1R in an IGF1R dimer contains six autophosphorylation tyrosine sites, labeled “Y” followed by the site’s position within the polypeptide chain (according to UniProt numbering). The kinase domains of each IGF1R subunit are labeled accordingly and encompass the region spanning positions 999 to 1274. Autophosphorylation and dephosphorylation of activated (IGF1-crosslinked) receptors in a dimer occur at the tyrosine sites indicated. Signaling proteins containing SH2 and/or PTB domains are recruited to their cognate tyrosine sites in a phosphorylated IGF1R dimer as indicated by the colored lines that begin at a tyrosine site and end at the protein that is recruited to that site.

We modeled IGF1-IGF1R interactions based on the harmonic oscillator (HO) mechanism proposed by Kiselyov et al. [44] and supported by subsequent structural analyses [47–49]. IGF1R molecules exist in pre-formed dimeric complexes, each of which contain two IGF1 binding pockets that are considered to be functionally equivalent (Fig 1A) [50]. Each binding pocket contains two dissimilar IGF1 binding sites, termed Site 1 (S_1_) and Site 2 (S_2_). A binding pocket can accommodate at most one IGF1 molecule, so a dimeric complex can simultaneously engage at most two IGF1 molecules. According to the HO mechanism, an IGF1 molecule first engages S_1_ or S_2_ in one of the two binding pockets of a dimer. Sites S_**1**_ and S_2_ are known to have differing affinities for ligand binding [47,51], such that there exists a primary route toward receptor crosslinking whereby the high affinity site is bound first, as well as a secondary route whereby the ligand binds the lower affinity site first [44]. After binding one site (most likely the high affinity site), the other IGF1 binding surface may then engage the remaining site in the pocket. The two sites in the pocket are crosslinked when both binding surfaces of one IGF1 molecule simultaneously occupy S_1_ and S_2_ (Fig 1B). Crosslinking stabilizes the active conformation, wherein transmembrane and intracellular regions of the receptor are close together [48,52], making the receptor competent for autophosphorylation of intracellular phosphotyrosine sites. When one pocket is crosslinked, the S_1_ and S_2_ sites of the other pocket are located too far apart to permit ligand engagement of both sites.

Recent crystallographic data have led to the proposal of an alternative mechanism for IGF1-IGF1R binding, which has been termed the induced fit mechanism [51]. In this picture of IGF1 binding, receptors are generally present in a closed conformation, wherein incoming ligand cannot interact with either S_1_ or S_2_ sites with high affinity. In contrast, the HO mechanism assumes that a proportion of receptors are present in an open conformation, with ligand capable of binding to either S_1_ or S_2_. Receptor activation via the induced fit mechanism is initiated when ligand interacts with the binding pocket in a metastable manner. The structural data suggest that this pre-complex includes IGF1 interaction with S_1_ and putative residues contained in S_2_. Once the pre-complex is formed, the receptor may relax and shift to an open conformation in which IGF1 can crosslink S_1_ and S_2_. The induced fit mechanism was proposed after analysis of crystal structures of unbound IGF1R as well as IGF1 in complex with S_1_ in IGF1R. However, the crystallization chaperone is derived from the monoclonal antibody (mAb) 24–60, which is known to inhibit IGF1 binding to IGF1R [51,53]. Therefore, it is unclear how the reported structures supporting the induced fit mechanism reflect physiological conditions, and further study is needed to determine the in vivo conformations of IGF1R important for interaction with IGF1.

For the models presented here, we consider that IGF1 molecules are capable of reversibly associating with and crosslinking one of the two binding pockets, modulating IGF1R receptor transition between inactive and active conformations. Ultimately, our model preserves the major features of both the HO and induced fit mechanisms, each of which entails two steps to achieve activation of IGF1R. In either mechanism, IGF1 initially engages IGF1R, either by binding one site (HO mechanism) or by forming a pre-complex (induced fit mechanism). Then, the ligand-receptor complex is stabilized in a conformation competent for autophosphorylation, either via completion of crosslinking of the bound IGF1 (HO mechanism) or by conformational change of ligand and receptor (induced fit mechanism).

**Fig 2:**
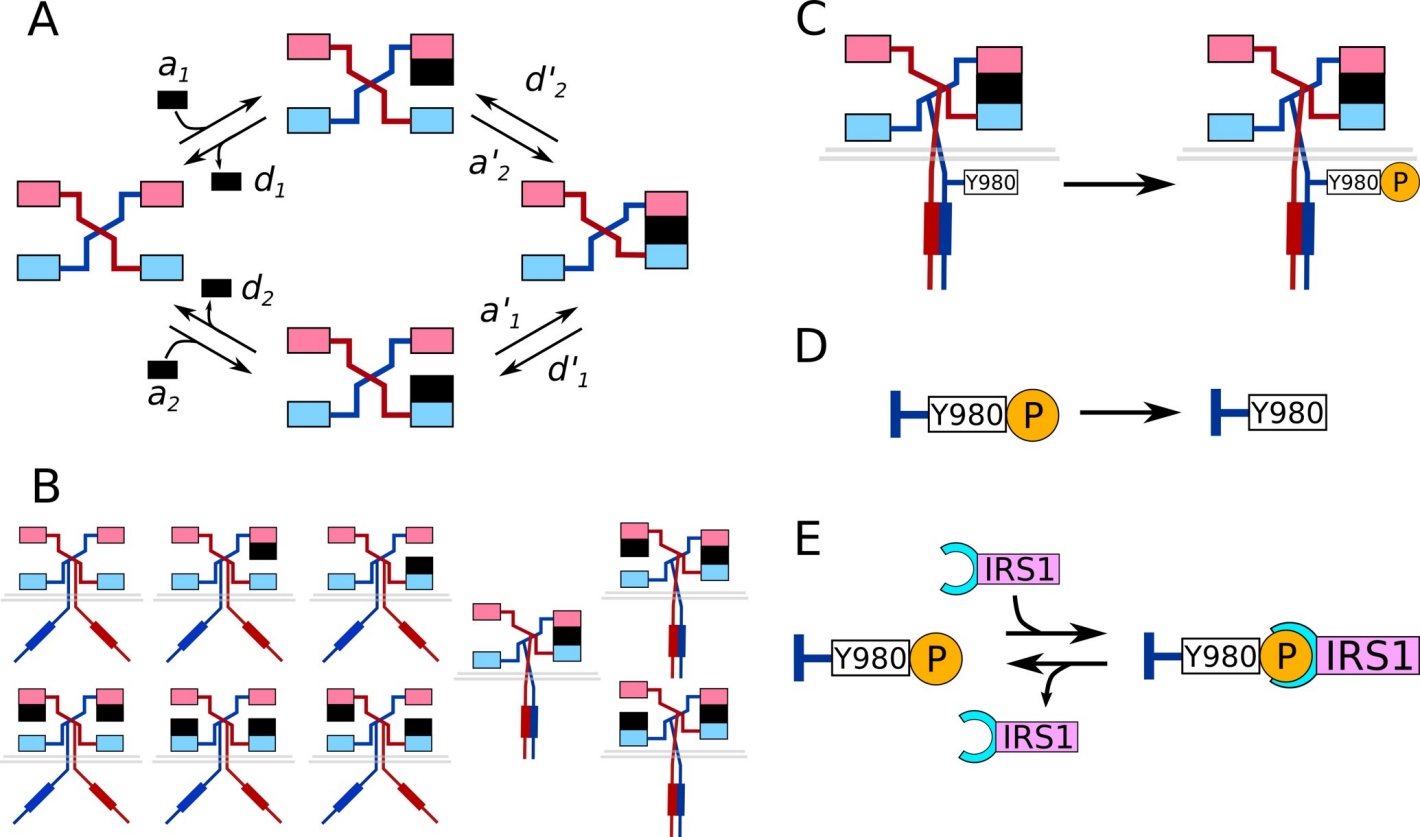
Model for IGF1 interaction with IGF1R. The model considers IGF1R to be present in preformed dimers, where each dimer contains two ligand binding pockets. Within each pocket, there are two sites, which we refer to as sites S_1_ (light pink) and S_2_ (light blue). S_1_ and S_2_ are bound by IGF1 (black rectangle) as signified by direct contact. Adjacent S_1_ and S_2_ sites are crosslinked when they are bound by the same IGF1 molecule, and, when crosslinking occurs, the kinase domains of the two receptor subunits come into contact. The IGF1R dimer is thus activated and can catalyze autophosphorylation. (A) The cyclic reaction scheme of IGF1-IGF1R binding presented here is derived from the model of Kiselyov et. al. [44]. (B) IGF1-IGF1R complexes that arise from the reactions of the cyclic reaction scheme. (C) Phosphorylation of one of the six IGF1R tyrosine sites, Y980. (D) Dephosphorylation of the Y980 site of IGF1R. (E) Recruitment of a representative IGF1R binding partner, IRS1 (pink structure), to phosphorylated Y980 via the IRS1 PTB domain (cyan structure), and subsequent dissociation.

IGF1R possesses six autophosphorylation sites: Y973, Y980, Y1161, Y1165, Y1166, and Y1346 (Fig 1B). Note that this list of sites does not correspond to sites considered in some previous studies [46,54], as we excluded Y1280 and Y1281. The literature suggests that Y1280 and Y1281 are not in fact autophosphorylated (as indicated in some databases), but rather, are important in coordinating binding to the surrounding serines [55,56]. Fig 1B illustrates the mapping of IGF1R signaling proteins to the phosphotyrosines that they are capable of binding. The possible IGF1-IGF1R interactions shown in Fig 2A give rise to nine distinct dimeric complexes, of which three represent crosslinked receptors (Fig 2B). Tyrosine phosphorylation (Fig 2C) and dephosphorylation (Fig 2D) are each modeled as a one-step process (i.e., a first-order reaction). For all six tyrosines, we apply the same pseudo first-order phosphorylation rate constant (0.5 s^-1^) and dephosphorylation rate constant (0.1 s^-1^). These simplifications are acceptable based on the common assumption that neither tyrosine kinases nor phosphatases are saturated. The concentration of IGF1R considered in the model is in the nM range; RTKs have been observed to become saturated when concentrations are in the μM range [57]. The half-life of a phosphorylated tyrosine residue is on the order of seconds [58], which indicates excess phosphatase activity. Once a tyrosine site is phosphorylated, it can recruit its respective SH2/PTB domain-containing binding partner(s) (Fig 2E). Dephosphorylation is only permitted if a site is not occupied by a signaling protein.

IGF1-IGF1R interactions are characterized by a cyclic reaction scheme with eight reactions (Fig 2A). The kinetics of forward (binding) reactions are characterized by the rate constants *a*_1_, *a*_2_, *a'*_1_, and *a'*_2_, where *a'*_1_, and *a'*_2_ characterize the rate of crosslinking of S_1_ and S_2_. The corresponding dissociation rate constants are *d*_1_, *d*_2_, *d'*_1_, and *d'*_2_. We take the view that the oscillatory movements of IGF1R dimers are spontaneous, meaning driven purely by thermal fluctuations without any dissipation of energy (e.g., no consumption of ATP). Thus, the IGF1-IGF1R cyclic reaction scheme must satisfy the principle of detailed balance [59,60]. In other words, the rate constants of the cyclic reaction scheme cannot be specified independently, and are related by the following constraint:

$$\left(\frac{a_{1}}{d_{1}}\right)\left(\frac{a\prime_{2}}{d\prime_{2}}\right)=\left(\frac{a\prime_{1}}{d\prime_{1}}\right)\left(\frac{a_{2}}{d_{2}}\right)$$ (1)

**Fig 3:**
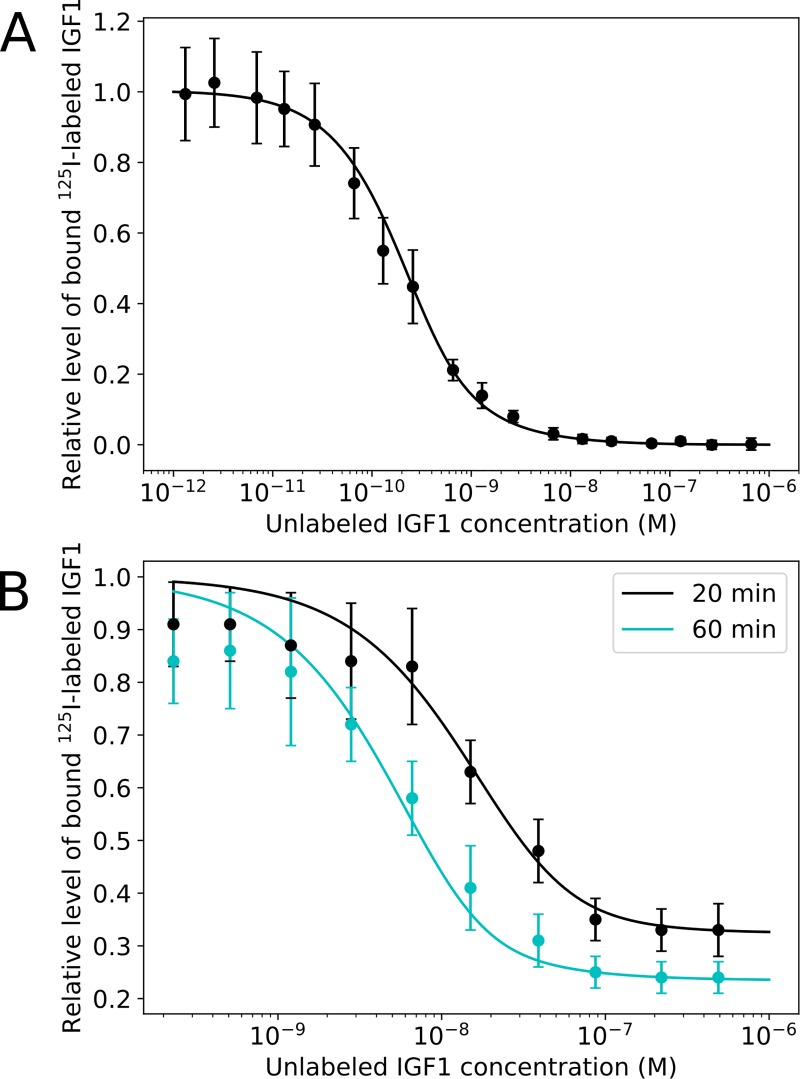
Model parameterization. Parameter values for IGF1-IGF1R binding were obtained by fitting to data from competition experiments and ligand dissociation experiments presented by Kiselyov et al. [44]. Experimental data are shown as points with error bars and the smooth curves were obtained via numerical simulation. For (A), we simulated to steady-state for a range of concentrations of unlabeled IGF1. For (B), we simulated a two-hour incubation with radiolabeled IGF1, then free ligand was washed away (i.e., the abundance of free IGF1 was set to zero), and we simulated the response to ligand addition for either 20 or 60 minutes, as indicated, for a range of concentrations of unlabeled IGF1.

To estimate the values of the rate constants, we used BioNetFit [61] and IGF1-IGF1R ligand dissociation data and equilibrium binding data presented in Ref. [44]. We estimated the values of *a*_1_, *a*_2_, *a'*_1_, *d*_1_, *d*_2_, *d'*_1_, and *d'*_2_. The remaining rate constant, *a'*_*2*_, was determined by the detailed balance constraint of Eq 1. We did not assume *d*_1_ = *d'*_1_ or *d*_2_ = *d'*_2_. Kiselyov et al. found improved fits without the assumption that these pairs of dissociation rate constants are equivalent, but concluded that the inclusion of the equivalency constraint only marginally detracted from the quality of the fit. We found that, with the inclusion of the detailed balance constraint, acceptable fits (χ^2^ ≤ 3.5) could not be achieved with *d*_1_ = *d'*_1_ and *d*_2_ = *d'*_2_. The curves generated with our best-fit parameter estimates are plotted in Fig 3. Note that the assignment of S_1_ and S_2_ is arbitrary, such that equivalent fits and simulation results are obtained when swapping the parameters of the two paths (i.e., swapping *a*_*1*_ with *a*_*2*_, *d*_*1*_ with *d*_*2*_, *a'*_*1*_ with *a'*_*2*_, and *d'*_*1*_ with *d'*_*2*_).

From our best-fit parameters in Table 1, we estimate site-specific *K*_*D*_ values to be 13 nM and 180 nM, comparable to the 9 nM and 490 nM estimated by Kiselyov et al. [44] and the value of 39 nM for IGF1 binding IGF1RΔβ (an ectodomain-only construct) measured by Xu et al. [51]. Furthermore, we estimated apparent *K*_*D*_ values using the simplification presented by Ullah et al. [62]. With this approach, the two-step pathway comprising initial ligand binding followed by crosslinking is approximated as a one-step reaction. We determined an apparent *K*_*D*_ value of 0.61 nM using the best-fit parameter set. This estimate is similar to the 0.12 nM reported by Kiselyov et al. [44] and subsequently used to fit an induced fit binding model in the study of Xu et al. [51].

**Table 1:** Comparison of parameter values for IGF1-IGF1R binding. We estimated rate constants (defined as indicated in Fig 2) by fitting to IGF1-IGF1R binding data [44] using the BioNetFit software package [61]. We calculated the 95% confidence interval for each parameter using a bootstrapping procedure (see Material and Methods). The parameter *a'*_*2*_ was taken to be given by Eq 1 and the values of the other rate constants.

	This Study			Kiselyov et al. [44]
Parameter	Estimate	Lower Limit	Upper Limit	Estimate
***a***_**1**_ **(M**^**-1**^**s**^**-1**^**)**	2.8×10^5^	2.3×10^5^	9.7×10^5^	3.5×10^5^
***d***_***1***_ **(s**^**-1**^**)**	5.0×10^−2^	1.1×10^−2^	0.13	3.2×10^−3^
***a***_***2***_ **(M**^**-1**^**s**^**-1**^**)**	1.5×10^4^	3.5×10^3^	2.2×10^4^	8.7×10^3^
***d***_***2***_ **(s**^**-1**^**)**	1.9×10^−4^	1.1×10^−5^	2.6×10^−4^	4.2×10^−3^
***a'***_**1**_ **(s**^**-1**^**)**	5.6×10^−3^	1.6×10^−4^	0.29	0.33
***d'***_***1***_ **(s**^**-1**^**)**	1.9×10^−5^	3.8×10^−7^	6.9×10^−4^	*d*_*1*_
***a'***_***2***_ **(s**^**-1**^**)**	52	0.45	5.7×10^4^	*a'*_*1*_
***d'***_***2***_ **(s**^**-1**^**)**	1.3×10^−2^	3.8×10^−3^	2.7×10^−2^	*d*_*2*_

The IGF1R model with a natural formulation of rules has tremendous combinatorial complexity, which presents a significant challenge when performing simulations. There are three possible configurations in which a receptor dimer can be crosslinked (Fig 2B). Every receptor monomer has six tyrosine sites, or 12 sites in each receptor dimer, each of which can be unphosphorylated, phosphorylated but unbound, or phosphorylated and bound to an SH2/PTB domain-containing protein. With nine possible configurations of IGF1 bound to IGF1R (Fig 2B), a coarse estimation of the total number of possible unique species (avoiding double counting of symmetrical species) is ≈ 9 × (3^12^ + 3^6^)/2 = 8×10^5^. It is impractical to generate and simulate the full reaction network between these species. To address this issue, we have optimized the formulation of rules, through a process we term *restructuration*, to obtain a rule set that implies an equivalent network with minimal state redundancy. We have outlined the approach used to restructure the IGF1R signaling model in Materials and Methods, and provided a detailed overview of restructuration in S1 File. After restructuring, the IGF1R model contains 577 species, or < 0.07% of the number of species implied by the natural formulation.

### IGF1R recruitment of signaling proteins in HeLa S3 cells

Which signaling proteins are most often recruited to IGF1R depends on many factors, including relative protein expression levels (S1 Table), relative binding affinities (S2 Table), and competition for each of the six autophosphorylation sites. We first analyzed signaling protein binding in a HeLa S3 cellular background, parameterized with proteomics data from Kulak et al. [63]. Out of 18 possible binding partners, only ITK and ZAP70 are not expressed in HeLa S3 cells (S1 Table).

**Fig 4:**
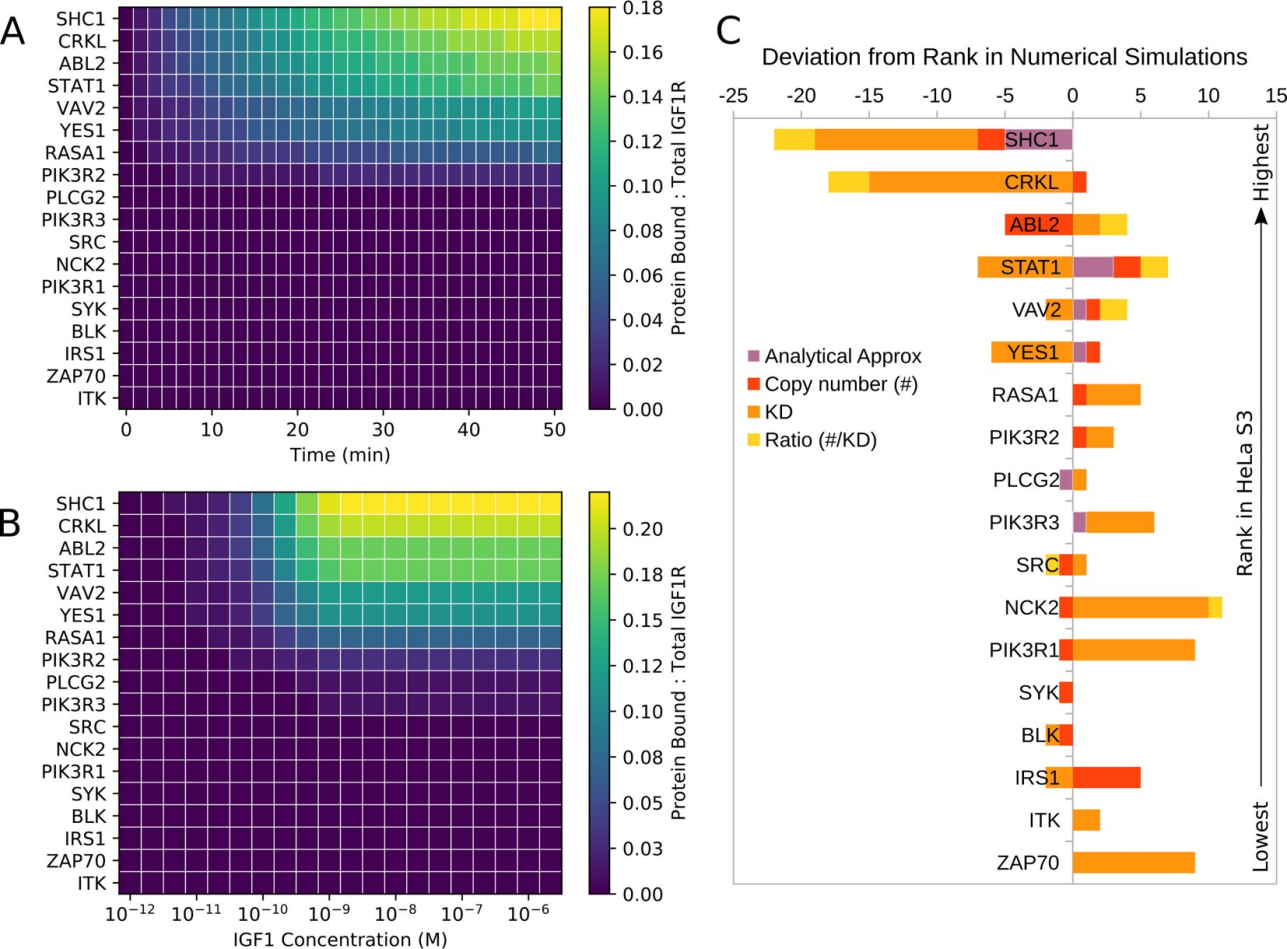
Predicted recruitment of signaling proteins to IGF1R in HeLa S3 cells. The scale represents the normalized amount of each of the indicated proteins bound in simulations of either (A) time-dependent recruitment at 1 nM IGF1 stimulation, or (B) steady-state recruitment at varying IGF1 doses. (C) Comparison of rank ordering of IGF1R binding partners in HeLa S3 cells across five prediction methods. The top (first) ranked protein indicates the protein predicted to be bound to IGF1R with the highest abundance. Bar length indicates the amount of deviation from the ranking obtained by numerical simulations for estimates obtained by the analytical approach, copy number, by K_D_, or the ratio of copy number to K_D_. A negative deviation indicates that the method estimated a lower rank (less binding) than the prediction from numerical simulations; a positive deviation indicates a higher rank (more binding) than in numerical simulations. Calculation and evaluation of rank ordering are described in Materials and Methods.

Fig 4A shows the model prediction for the temporal recruitment of SH2/PTB-domain containing proteins in HeLa S3 cells in response to 1 nM IGF1 stimulation. Fig 4B shows the steady-state recruitment at various doses of IGF1 stimulation. Simulations indicate strong recruitment (17–22% of IGF1R bound at steady-state with 1 nM stimulation) of SHC1, CRKL, ABL2, and STAT1 and modest recruitment (7–12% of IGF1R bound) of VAV2, YES1, VAV2, and RASA1.

The adapter protein SHC1 is predicted to be the most highly recruited protein in HeLa S3 cells. On IGF1R, SHC1 binds only to pY980, a site that also recruits IRS1 and ZAP70. As ZAP70 is not expressed in HeLa S3 cells, the only competitor for SHC1 in this cellular background is IRS1. The strong recruitment of SHC1 is due to both its high copy number (10^5^/cell) (two orders of magnitude higher than that of IRS1, see S1 Table) and its strong affinity for this site (20-fold stronger than IRS1, see S2 Table).

Next, we consider STAT1, CRKL, and ABL2, which all display strong IGF1R recruitment. STAT1 and CRKL are the two most abundant binding partners in HeLa S3 cells, with relatively high concentrations (10^5^ copies per cell), whereas ABL2 is the ninth most abundant signaling protein, with an abundance on the order of 10^4^ copies per cell. STAT1 binds pY1161, which also recruits PIK3R3, SRC, YES1, SYK, and BLK. Despite these five competitors, recruitment of STAT1 is strong because the five competing proteins have relatively low expression levels (S1 Table). Like STAT1, CRKL competes with multiple proteins that are expressed at relatively low levels for binding to pY1166 and pY973. High recruitment of ABL2 can be explained by the strong affinity of ABL2 for pY973, which is approximately 40-fold greater than that of CRKL.

### Predictions from simulations vs. predictions from simple ranking approaches

We observe that the rank order predicted by numerical simulations is distinct from that obtained through ranking by *K*_*D*_, copy number, or the copy number/*K*_*D*_ ratio (Fig 4C), suggesting that competition, protein abundances, and binding affinities all contribute to the relative recruitment of downstream partners in an unintuitive manner. We further evaluated whether simple methods could return the same predictions obtained from simulations by deriving analytical expressions of equilibrium binding (S3 File, Eq. S1–S9). These expressions roughly estimate how the rank ordering of binding partners is related to factors affecting binding partner recruitment, including copy number and binding affinity. This approach is similar to that proposed by Maslov and Ispolatov [64].

To derive analytical expressions for estimating recruitment, we exploit a key simplification that assumes each IGF1R binds a single partner at a time. In the rule-based model, we allow for recruitment of multiple binding partners to different pY sites, which is consistent with the findings of a recent study where multi-site phosphorylation of EGFR was directly observed through and single-molecule fluorescence microscopy [65]. However, another study using related technology found that ligand-activated EGFR is infrequently multi-phosphorylated for the sites probed [66]. Thus, RTKs may in some cases bind only a single downstream signaling partner at a time, so an acceptable analytical approximation for these cases may be obtained by the application of this assumption.

The concentration of protein bound to IGF1R can be expressed as a function of the total protein concentration, the free, active IGF1R concentration, and the measured binding affinity between the protein and the receptor. Because each active receptor is assumed to bind only one signaling protein at a time, this relationship can be represented by the following equation (derived in S3 File as Eq. S9):

$$X_{i}^{Bound}\approx R\cdot \frac{X_{i}^{Tot}}{K_{i}+R}$$ (2)

In Eq 2, $X_{i}^{Bound}$ represents the concentration of the *i*^*th*^ protein species that is bound by IGF1R, $X_{i}^{Tot}$ represents the total concentration in the system of the *i*^*th*^ protein species, *K*_*i*_ is the apparent dissociation constant for partner *X*_*i*_, and *R* is the concentration of free, active IGF1R. To account for competitive binding, we need to calculate the free active IGF1R concentration, *R*, from the following balance equation for the total active IGF1R (*R*^*Tot*^), see S3 File. Eqs 2 and 3 provide a means to rank IGF1R binding partners.

$$R\left(1+\sum_{i=1}^{N}\frac{X_{i}^{Tot}}{K_{i}+R}\right)=R^{Tot}$$ (3)

There are six inconsistencies in rank order when comparing predictions from numerical and analytical methods (Fig 4C). Differences between the analytical and numerical rankings are minimized as the concentration of free active IGF1R in the analytical model increases, which suggests that these discrepancies arise from the assumption that each active RTK can bind only one binding partner at a time, described in more detail in S3 File. We also compared quantitative predictions from numerical simulations to those from the analytical approximation (S3 Fig). There is a strong positive correlation between the steady-state number of bound molecules predicted with each of the methods (Pearson’s *r* = 0.93 with a *p* value of 10^−8^ for HeLa S3, obtained using R software’s cor.test function), which provides support for use of the analytical approach for generating rough predictions. However, the degree of agreement between the approaches depends on the specific protein considered. Overall, we conclude that it is difficult to capture the predictions of the rule-based model solely via simple metrics like copy number or binding affinity, or even with our analytical expressions.

### Determining rankings of IGF1R recruitment of signaling proteins across diverse cell lines

We investigated whether competition among the signaling proteins could be influenced by cell type-specific differences in protein copy number. In addition to the HeLa S3 cell model discussed above, we parameterized 44 other models to represent diverse cell types. Eleven human cell lines were studied by Geiger et al. [67]. An additional 31 models were formulated for cell lines within the NCI-60 panel derived from human tumors and maintained by the National Cancer Institute. We developed models for only the NCI-60 cell lines in which IGF1R was detected [68]. HeLa Kyoto proteomics data were from Hein et al. [69]. We also considered one non-human cell line, the immortal murine myoblast cell line C2C12 [70], to allow for comparison with available binding data [71].

**Fig 5:**
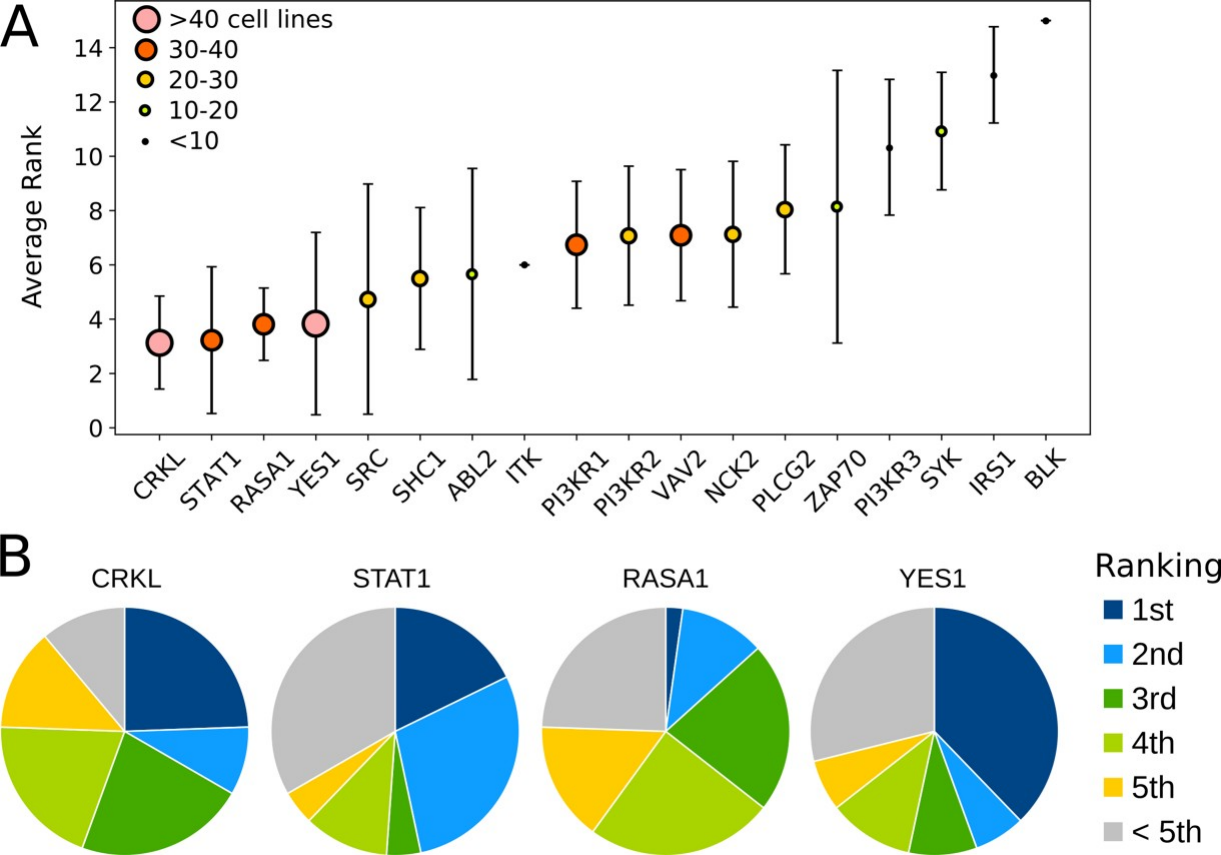
Summary of rank ordering of IGF1R binding partners across 45 cell line-specific models. (A) The average rank across all models is shown for each binding partner. Error bars indicate the standard deviation in rank. The size of a data point indicates the number of cell lines in which the corresponding protein was expressed, as shown in the legend. (B) For the four binding proteins with the highest average rank, we show the percentage of cell lines in which the protein was ranked first, second, third, fourth, fifth, or lower than fifth.

For each cell line model, we simulated the steady-state pattern of recruitment of signaling proteins to IGF1R in response to stimulation with 1 nM IGF1. Across all cell lines, the most highly recruited proteins were predicted to be CRKL, STAT1, RASA1, and YES1 (Fig 5). The ranking from numerical simulations for each cell line is provided in S2 File along with predicted ranks from four simple ranking metrics: analytical approximation, copy number, equilibrium dissociation constant (*K*_*D*_), and copy number divided by *K*_*D*_.

YES1 was the top-ranked binding partner in 38% of cell lines (Fig 5B). It has been suggested that YES1’s SH2 domain is remarkably promiscuous [45], such that the high recruitment predicted by our model may not accurately reflect recruitment in vivo. YES1 and other proteins in the SRC class of tyrosine kinases have been found to be highly connected, a factor which may contribute to oncogenic potential [46]. However, promiscuity does not necessarily indicate that YES1 is not a legitimate IGF1R binding partner. Indeed, the literature offers evidence that YES1’s SH2 domain does exhibit specificity. For example, YES1 reportedly binds to pY1161 and pY1165 with *K*_*D*_ values of approximately 0.9 μM, but was not strongly recruited to the other IGF1R tyrosine sites [46]. Similarly, YES1 is recruited to three of the eight sites in the INSR with *K*_*D*_ values less than 1 μM. In ErbB-family RTKs, including EGFR, ErbB2, ErbB3, and ErbB4, YES1 was either not detected as an interacting partner or bound weakly, with a *K*_*D*_ greater than 2 μM [11]. These observations suggest that YES1 is a bona fide binding partner relevant for IGF1R signaling, but further investigation is needed.

Hanke et al. [71] analyzed protein recruitment to IGF1R in the mouse myoblast cell line C2C12 via a pulldown assay. To our knowledge, Hanke et al.’s study provides the best data available to aid in evaluating predictions from our IGF1R binding model. Their procedure, in which peptides (15-mers) spanning each of the murine IGF1R phosphotyrosine residues were incubated with cell lysate, allows binding partners to compete for individual phosphotyrosines. The ratios of proteins detected with and without each phosphorylated tyrosine were reported. Unfortunately, this normalized recruitment data cannot provide a ranking of the most highly recruited binding partners, especially because each phosphotyrosine was considered separately. However, the pulldown data does provide a picture of detectable binding partners, which can be compared to model predictions. The most highly recruited protein in C2C12 simulations was RASA1, a GTPase-activating protein (GAP) for RAS. This protein was also detected in the pulldown assay, with a reported 8-fold increase in binding between phosphorylated and unphosphorylated Y1283 in murine IGF1R. Other proteins that were predicted to bind in simulations and detected by the pulldown assay include the PI3K regulatory α and β subunits (PIK3R1 and PIK3R2), which displayed 15-fold and 9-fold increase in binding, respectively, to pY1352 over Y1352.

Differences between the C2C12 model predictions and pulldown assay results arise because our models were designed to include only detectable interactions between human IGF1R and SH2/PTB domain-containing proteins. We kept binding affinities consistent across all cell line-specific models, including in simulations with the murine C2C12 cell line, an assumption based on previous findings that binding affinities are often evolutionarily conserved across eukaryotic organisms [72]. However, this approach resulted in exclusion from the model of many binding partners detected in the murine pulldown assay. For instance, human PLCG2, not PLCG1, has a detectable affinity for IGF1R [45,46], so only PLCG2 was included as a potential binding partner in the model, yet expression of PLCG2 in C2C12 was negligible [70]. Therefore, the model parameterized with protein copy numbers for C2C12 cannot predict binding of either PLCG1 or PLCG2. Likewise, GRB2, SHP2, PIK3CA, and PIK3CB were captured in the murine pulldown assay, but were not included in the models due to lack of detectable IGF1R binding in humans [45,46]. Thus, in this case human binding affinity data are not sufficient for predicting all binding partners in mouse myoblasts. The discrepancies between model predictions and experimental evidence could perhaps be rectified if *K*_*D*_ data for murine SH2/PTB domain interactions with IGF1R were to become available. However, pulldown assays are not ideally suited for the task of evaluating rank order predictions. One intriguing experimental approach that could be useful for this purpose is mass-spectrometry (MS)-based phosphoproteomics, which was recently used by Jadwin et al. [17] to measure phosphorylation of specific phosphosites in full-length EGFR. Most pY sites are bound to an SH2/PTB-domain containing protein, so quantifying which sites are highly phosphorylated could validate predictions of which partners are highly bound.

**Fig 6:**
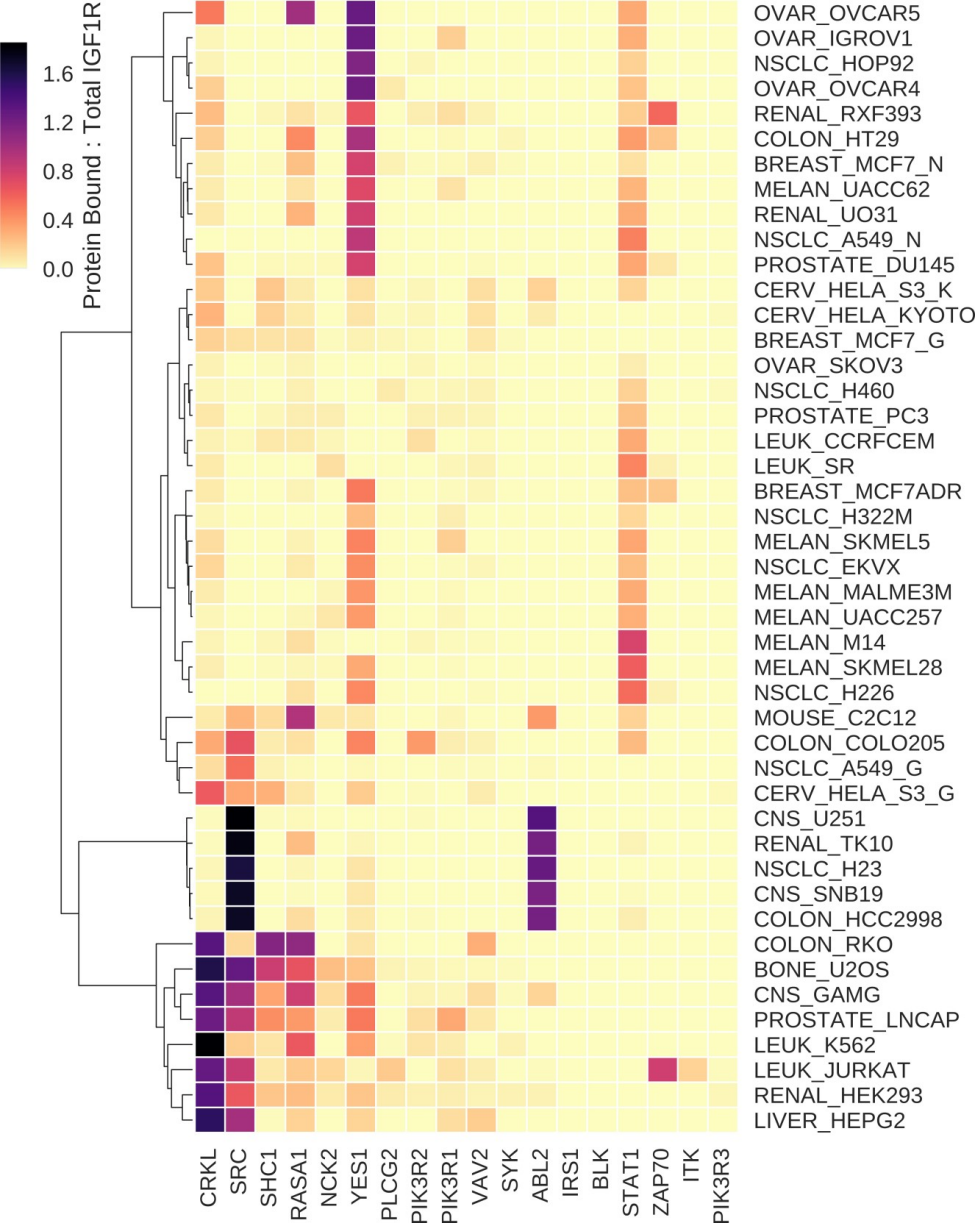
Hierarchical clustering of cell lines according to predicted signaling protein recruitment. Heatmap colors indicate the ratio, derived from simulation results, of the number of bound molecules of a given protein (corresponding to protein name on the x-axis) divided by the total number of IGF1R molecules. As multiple copies of a protein can be bound to one IGF1R molecule, the ratio can be greater than one. Cell lines indicated (on the y-axis) include the tissue of origin or type of cancer followed by an underscore and the cell line name. OVAR = ovarian cancer, NSCLC = non-small cell lung cancer, MELAN = melanoma, CERV = cervical cancer, CNS = central nervous system cancer, LEUK = leukemia. For those cell lines evaluated in multiple studies (A549, HeLa S3, and MCF7), the proteomics data from Geiger et al. is indicated with “_G” following the cell line name, the data for the NCI-60 panel from Gholami et al. with the suffix “_N”, and the data from Kulak et al. with “_K”.

To determine whether cell line-specific IGF1R recruitment is indicative of tissue of origin, we performed hierarchical clustering to group cell lines according to similarity in protein recruitment profiles (Fig 6). In some cases, we do observe that similar tissue types clustered together. The HeLa S3 cell lines profiled by Kulak et al. [63] and the HeLa Kyoto cell line profiled by Hein et al. [69] were grouped together. Five out of six melanomas were co-clustered: M14, SKMEL28, UACC257, MALME3M, and SKMEL5. All of these cell lines had high recruitment of STAT1 and moderate recruitment of YES1. We also observe co-clustering of ovarian cancer cell lines OVCAR5, IGROV1, and OVCAR4, which had high predicted recruitment of YES1.

In examining the cell line clusters, we note that the source of the data contributes to similarity in predicted recruitment profiles. Eight out of the 11 cell lines profiled by Geiger et al. [67] are grouped together: RKO, U2OS, GAMG, LnCap, K562, Jurkat, HEK293, and HepG2. These cell lines stem from disparate tumor or tissue types, but they were all predicted to have very high recruitment of CRKL (more than one copy of CRKL bound to each molecule of IGF1R) coupled with moderately high recruitment of either SRC, SHC1, or RASA1. Similarly, of the seven cell lines that were profiled with higher coverage by Gholami et al. [68], four were clustered together: the ovarian carcinoma SKOV3, non-small cell lung cancer H460, the prostatic adenocarcinoma PC3, and the acute lymphoblastic leukemia CCRFCEM. This result indicates that, on the basis of IGF1R signaling, these four cell lines are predicted to be more similar to one another than to cell lines from the same tissue type. However, we suspect that clustering is highlighting an artifact in the data. Most of the NCI-60 cell lines have only low coverage proteomics data available; therefore, copy numbers derived from these data may not accurately represent the protein abundance profiles of these cell lines. Thus, while it is clear that IGF1R binding partner recruitment is influenced by protein copy number, the quality of the proteomics data used to parameterize the models is critical in using simulations to generate reliable predictions. We will set aside concerns with the quality of the available proteomics datasets for the sake for demonstrating the capabilities of cell line-specific models.

### Correlations and anti-correlations in IGF1R-recruitment of proteins

**Fig 7:**
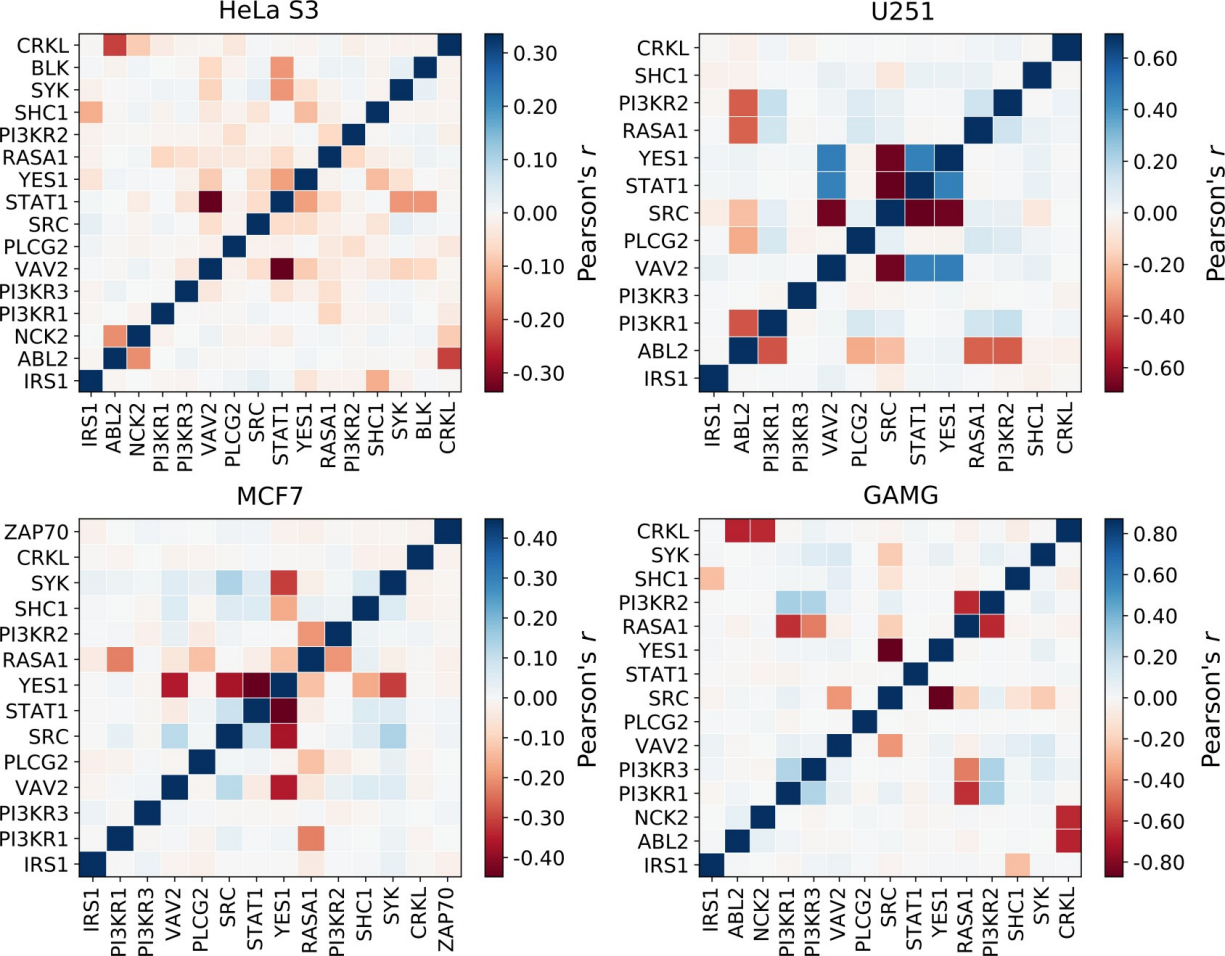
Correlation matrix among the signaling proteins for IGF1R recruitment. The color map shows the Pearson’s correlation coefficient among pairs of proteins studied in the population-level models for HeLa S3, U251, MCF7, and GAMG cell lines. Red indicates a negative correlation, blue a positive correlation, and white no correlation.

To further interrogate the mutual dependency among signaling proteins, we developed population-level models for IGF1R signaling. The population-level models are designed to account for natural cell-to-cell variability in protein levels present in a clonal population of cells [73]. This analysis produces a distribution of recruitment profiles across 5,000 cells in each simulated population, thereby allowing us to ascertain whether the recruitment of any pair of binding proteins is correlated. Fig 7 shows a matrix of the correlations between each pair of binding partners for the HeLa S3, U251, MCF7, and GAMG population-level models (correlations for all other cell lines are in S4 and S5 Figs). The degree of positive or negative correlation between any two proteins may arise from direct competition for receptor binding or via indirect effects, as discussed below.

In the HeLa S3 model, the moderate negative correlation (Pearson’s *r* = -0.34) between VAV2 and STAT1 arises from their direct competition for pY1161. Both proteins are present at relatively large and comparable abundances (10^5^ copies per cell), bind only to pY1161 with similar affinities, and therefore effectively antagonize each other. Other cell lines with a negative correlation between VAV2 and STAT1 included the leukemias CCRFCEM and SR, the melanoma M14, the non-small cell lung cancers H226 and H460, and the prostatic adenocarcinoma PC3. Negative correlations are also observed with discrepancies in binding affinity or copy number between competing pairs. The weak negative correlation between CRKL and ABL2 in HeLa S3 cells (Pearson’s *r* = -0.23) can be explained by competition for binding to pY973; these proteins have similar abundances, but ABL2 binds pY973 with approximately 40-fold higher affinity.

Slight variations in protein copy number contribute to differing patterns of correlations across cell lines. Cell line-specific differences are especially evident when considering competition for pY1161, to which nine different signaling proteins can bind. The pair of proteins displaying high negative correlations in the largest number of cell lines was YES1 and STAT1, which compete for binding pY1161 and were negatively correlated in 19 cell lines, including MCF7 (Fig 7). The average Pearson’s *r* for YES1/STAT1 was -0.55±0.18 across these 19 cell lines. In each of these cell lines, the abundances of YES1 and STAT1 were within an order of magnitude of one another, and other proteins that bind pY1161 were either not expressed or expressed at a low level in comparison to YES1/STAT1. In 14 cell lines, where STAT1 had low expression, and SRC expression was comparable to that of YES1, strong negative correlations between YES1 and SRC were evident, with an average Pearson’s *r* = -0.67±0.21 across these 14 cell lines (see correlations for MCF7 and GAMG in Fig 7).

Interestingly, positive correlations between binding partners are observed in several of the cell line-specific population-level simulations, despite there being no cooperative binding explicitly considered in the models. These positive correlations arise through an indirect mechanism. In four cells lines, including the gliomas U251 and SNB19, the renal cell carcinoma TK10, and the colorectal carcinoma HCC2998, a pattern of positive correlations (Pearson’s *r* ≥ 0.4) was identified for every pairwise combination of STAT1, VAV2, and YES1 (see Fig 7 for U251). Furthermore, we identified strong negative correlations between SRC and STAT1, VAV2, and YES1 in these cell lines. These four proteins compete for binding to pY1161, but in these cellular backgrounds, SRC is expressed much more highly (10^7^ copies per cell) than STAT1, VAV2, or YES1 (10^2^–10^6^ copies per cell). Thus, stochastic decreases in expression of higher abundance competitors results in a net increase in the opportunity for binding lower abundance proteins, observable as a positive correlation between lower-abundance binding partners. In other words, positive correlations arise when a pair or group of proteins are similarly affected by a mutual competitor.

## Discussion

In cell signaling, many receptors, including IGF1R, are capable of activating multiple signaling pathways in response to a common input signal. Mathematical modeling provides an avenue for predicting which subset of possible interactions are more likely to occur in a combinatorially complex network [74]. Because of the wealth of empirical data available, IGF1-IGF1R signaling represents an ideal case for study of competition in cell signaling. Here, we developed mechanistic models of IGF1R signaling to provide a method for determining how competitive protein interactions play a role in early events in receptor-mediated signaling. Our analysis shows that differences in signaling protein expression can be expected to impact the rank order of protein recruitment. A protein with higher abundance and lower binding affinity in a given cellular background can outcompete other proteins with lower concentrations and higher affinities. Thus, a given extracellular input can preferentially activate different cellular functions in diverse cellular backgrounds.

We adopted the rule-based modeling approach to develop models of IGF1R signaling. Given the large reaction network size implied by the rules as naturally formulated, simulation was a critical challenge. We addressed this challenge through model restructuration, meaning optimization of rule formulation so as to obtain a smaller rule-implied reaction network size without introducing approximations. We applied three strategies to restructure the model: decoupling, bunching, and scaling, the first two of which are related to previously described methods of model transformation (see S1 File). Through these fairly straightforward transformations, we were able to enumerate equations describing IGF1-IGF1R binding and signaling protein recruitment that could be efficiently integrated with an ODE solver. Similar strategies could be useful in simulating similar biochemical systems.

We demonstrated the difficulty of using naive prediction methods, such as copy number, binding affinity, or simple analytical expressions, to predict even the rank order of signaling protein recruitment. This is important, because it is still tempting to draw conclusions about recruitment profiles or important drug targets using measurements of only binding affinity or only RNA/protein abundance. Indeed, it is not unheard of in the literature to present recruitment profiles derived solely from *K*_*D*_ values [46,54], or to profess a phenotypic impact based on omics data [68]. While speculations based on simple metrics are not necessarily incorrect, our results show that a more detailed model exhibits nuances even in steady-state behavior that simple ranking metrics cannot recapitulate. Therefore, to best decipher early events in receptor signaling, it is recommended to build and simulate mechanistically-detailed models that incorporate experimentally determined parameters and/or are parameterized by fitting to experimental data. Mechanistic models of receptor signaling allow for consideration of the impacts of competition for binding phosphotyrosine residues, differences in binding affinity between SH2/PTB domains and pY sites, and differences in signaling protein abundance.

We propose these models as reasonable first approximations of early events in IGF1R signaling. Our predicted rankings can be justified because we expect all signaling proteins to have a comparable number of alternate binding partners. The study of Koytiger et al. [46] shows that each of the SH2/PTB domain-containing proteins considered here could bind an average of 76±35 phosphotyrosine sites in RTKs, with a range between 28 for VAV2 and 131 for SYK (excluding IRS1, which was only detected to bind INSR). However, in the scenarios simulated here, we are considering a case where IGF1 is the only input stimulus, such that other RTKs are not activated and therefore not competing for the recruitment of these binding partners. We further evaluated the number of alternate binding partners by querying the String Database [75] for known interactions, and found that each of the IGF1R binding partners participates in a highly connected network, with an average of 43±9 edges in a network comprising one signaling protein and its ten closest partners (minimum of 27 edges in the BLK network and maximum of 55 for the PIK3R1 and IRS1 networks). A number of the alternate partners interact with multiple IGF1R signaling proteins. After longer durations, differences in the number of possible alternate binding partners will impact the rank order of binding, as will differences in copy number of alternate binding partners, and differences in binding affinity for various interactions [76]. Given the extensive crosstalk present in metazoan signaling networks [77], we expect that additional layers in the signaling network will maintain the same degree of complexity, such that the need for modeling and numerical simulation only increases as higher order effects are considered.

Elevated IGF1R signaling has been implicated in many cancers, but thus far strategies to inhibit IGF1R have proven ineffective [78–80]. For instance, phase II or III trials evaluating the use of the anti-IGF1R mAbs ganitumab, dalotuzumab, and figitumumab were halted after showing no discernible anti-tumor activity or survival benefit [79]. Tyrosine kinase inhibitors (TKIs) such as linsitinib are able to inhibit IGF1R as well as the INSR, leading to toxicities from metabolic complications such as hyperglycemia [81]. Prolonged treatment of breast cancer with IGF1R TKIs has also been observed to promote resistance via activation of an alternate RTK, TYRO3 [80,82]. One burgeoning strategy is to target the IGF1R network rather than the receptor [80,83]. Simulations of our models could provide a rationale for selecting targets in the network that are more likely to be successful for treating cancers driven by IGF1R signaling. Here, we demonstrated the capabilities of the IGF1R model by developing cell line-specific models that used proteomics data available from diverse human cancer cell lines, including leukemias, melanomas, and breast, colon, prostate, renal, lung, bone, central nervous system, cervical, and ovarian cancers. Depending on the protein abundance profiles of these cell lines, our models generated predictions of the downstream information relays that would be preferentially activated. Many FDA-approved cancer drugs inhibit signaling proteins either directly recruited to IGF1R or downstream of the recruited proteins; thus, as different binding partners are more highly recruited in different cellular backgrounds, the most effective molecularly targeted drugs would likewise be cell type- or tumor-specific. For example, CRKL was predicted to be the most highly recruited protein across the cell lines evaluated here. CRKL activates MAPK signaling as well as AKT signaling via PI3K [84,85], so we hypothesize that an effective approach against many cancers might be to use a combination of AKT and/or ERK inhibitors. Treatment strategies could be adjusted for tumors displaying rare recruitment profiles, such as the highly ranked recruitment of PLCG2 predicted for the non-small cell lung cancer H460. Looking downstream of PLCG2 in the KEGG pathway database [86], we hypothesize that a tumor with high recruitment of PLCG2 could be successfully treated with combinations of COX-, ERK-, and PKC-inhibitors. Another potential strategy would be to use an IGF1R mAb or TKI in combination with drugs targeting molecules downstream of the most highly ranked binding partners.

Interestingly, our models do not predict highly ranked recruitment of the insulin receptor substrate 1 (IRS1), a canonical binding partner of INSR and IGF1R [87,88]. The average predicted rank of IRS1 recruitment across cell lines was 13±1.8 (Fig 5A). IRS1 competes primarily with SHC1 for binding to phosphotyrosine sites in the juxtamembrane region of the receptors. Although the *K*_*D*_ for IRS1 binding to IGF1R is not well-established, the PTB domain of IRS1 has an affinity for INSR phosphosites that is approximately 20-fold weaker than that of SHC1’s PTB domain [89], and protein copy numbers for IRS1 tended to be two to three orders of magnitude lower than those of SHC1 (S1 Table). Therefore, our models overwhelmingly predict recruitment of SHC1 over IRS1. How then, might IRS1 be recruited to IGF1R and INSR? One explanation could be that the ratio of SHC1 to IRS1 affinity for INSR is not in fact preserved for IGF1R. The amino acid sequences of INSR and IGF1R are not identical in the regions proximal to the respective tyrosines recruiting IRS1 and SHC1, which could promote differences in binding affinity. Disparity in the ratio of SHC1 to IRS1 affinity for these similar receptors could be a mechanism for divergence in the biological function of these receptors. Indeed, IRS1 could actually bind IGF1R with extraordinarily strong affinity, in the nanomolar range, as has been reported in one study employing surface plasmon resonance technology [90]. An alternate explanation for the lack of IRS1 recruitment predicted by our models might be attributed to the role of IRS1’s pleckstrin homology (PH) domain, which has been shown to be important for receptor recruitment [91]. The mechanism by which the PH domain contributes to recruitment is somewhat ambiguous; only about 10–15% of human PH domains are believed to bind phospholipids with strong affinity [92], and other evidence suggests that the IRS1 PH domain binds negatively charged regions of proteins [91]. Nonetheless, it is generally agreed that PH domains enhance membrane localization [93,94], so it is conceivable that IRS1’s PH domain leads to a higher effective concentration of IRS1 near the juxtamembrane region of receptors, sufficient to enable IRS1 to compete with cytoplasmic SHC1.

Surprisingly, we observed that the study from which proteomics data were taken was predictive of clustering of results from cell line-specific model simulations. This finding was concerning, because we would expect binding partner recruitment to be influenced by cell type, and not by the lab collecting the data. The models here were parameterized with shotgun proteomics data, which are evidently not well-suited for this purpose. Issues with shotgun proteomics data could emerge from biases introduced during sample preparation, inadequate coverage, or a limited ability to accurately quantify lower-abundance peptides [95–97]. Our approach here unintentionally serves as an example of how batch effects in proteomics data can be identified. By fitting data to a common model and clustering results, it becomes possible to identify datasets that are potentially problematic. Going forward, mechanistic modeling efforts may benefit from the increasing availability of data from targeted proteomics methods, such as that generated by Shi et al. [98]. Targeted proteomics allows for higher-confidence quantification of a selected subset of proteins, with higher sensitivity and reproducibility than shotgun proteomics [96,99]. To the best of our knowledge, such data are not yet available for IGF1R.

The idea that protein expression variability can significantly impact signal transduction has been previously proposed, including in studies of the EGFR, ErbB1, AKT, and ERK signaling networks [98,100–102]. Our analysis potentially explains the seemingly redundant wiring of networks with multiple signaling pathways, as follows. The models presented here provide a testable hypothesis that a receptor does not discriminate among potential downstream signaling pathways. Rather, pathway selectivity is the consequence of expression levels of recruited signaling proteins and the competition among them for receptor binding domains. Therefore, gene regulatory mechanisms, by affecting protein copy numbers, can modulate the function of cell signaling networks.

## Materials and methods

### Formulation of a generic model

We first developed a generic model for early events in IGF1R signaling that includes the following processes: IGF1 binding to IGF1R, autophosphorylation of IGF1R, dephosphorylation of phosphotyrosine sites, and reversible recruitment of signaling proteins with SH2 and PTB domains to IGF1R via interactions with phosphotyrosines.

The generic model was specified using the BioNetGen language (BNGL) [30]. All model specification files are available in S1 Compressed/ZIP File Archive. Rules were written for binding interactions between six phosphorylated tyrosines in IGF1R and the SH2/PTB domains of IGF1R binding partners characterized in the studies of Gordus et al. [45] and Koytiger et al. [46]. The following is an example of a rule for the binding of the PTB domain of one such binding partner, SHC1, to phosphorylated tyrosine residue 980 of IGF1R:

SHC1(PTB)+IGF1R(Y980~P)<->SHC1(PTB!1).IGF1R(Y980~P!1) ka,kd

The above rule includes two rate constants, one for binding (ka) and one for unbinding (kd), where mass-action kinetics are assumed. The kinase domain of each IGF1R monomer in a dimer was considered to be active if the dimer was crosslinked by IGF1. Phosphorylation and dephosphorylation of the receptor were modeled as first-order reactions.

We use the HUGO Gene Nomenclature Committee [103] approved gene symbols when referring to signaling proteins, where here the gene symbol in uppercase, non-italic font indicates a gene product. *K*_*D*_ values for binding of signaling proteins are from Gordus et al. [45] and Koytiger et al. [46], with the exception of IRS1, for which no detectable IGF1R binding was found in the aforementioned studies. As there is much evidence to suggest that IRS1 does interact directly with IGF1R [87,88], we approximated a *K*_*D*_ value based on quantification of interactions with INSR. Farooq et al. [89] found that the affinity of SHC1 recruitment to INSR at pY972 was approximately 20-fold stronger than that of IRS1. We assumed the same relation holds between SHC1 and IRS1 binding to IGF1R’s pY980, and set a *K*_*D*_ for IRS1 equal to 20-fold higher than that of SHC1 binding. Association rate constants (*k*_*a*_) for signaling protein binding were set to 1×10^6^ M^-1^s^-1^, and dissociation rate constants were calculated using the formula *k*_*d*_ = *k*_*a*_×*K*_*D*_. Some proteins have multiple SH2 domains reported as binding to the same tyrosine site, such as the two SH2 domains in PIK3R2 reported to bind pY1346 [46]. For these proteins, we assumed that the domain with the higher binding affinity would dominate binding and accordingly used the lower of the *K*_*D*_ values in the model. We do not consider the possibility of two-point attachment of proteins with multiple SH2/PTB domains to adjacent phosphotyrosines in IGF1R.

As a simplification, we do not consider receptor trafficking. The ligand dissociation curves to which we fit our model were obtained at low temperatures, which is expected to reduce recycling [44].

The models formulated in this study are not intended to capture the dynamics of later IGF1R signaling events. We do not consider any interactions between the binding partners of IGF1R themselves, nor do we attempt to predict the activation and subsequent activity of the binding partners after recruitment to IGF1R.

### Model restructuration

We used three strategies to restructure the natural model formulation and reduce state redundancy: decoupling, bunching, and scaling (S2 Fig). These strategies are described in further detail in S1 File.

1. *Decoupling*. The state of each phosphotyrosine site can be decoupled from the others because the phosphorylation dynamics of each site are assumed to be independent. If the state of one tyrosine site is not affected by the states of other sites, we can consider each of the six individual tyrosine sites as separate entities.

2. *Bunching*. The configurations of the S_1_ and S_2_ binding sites in a dimeric IGF1 receptor can be considered together as one binding pocket. Bunching the S_1_ and S_2_ sites is acceptable because the possible states for one site depend on the state of the other site in the pocket; both sites cannot simultaneously be bound to separate ligands. Specifically, the configuration of the S_1_-S_2_ binding pocket can either be 1) both sites are free, 2) one of the S_1_-S_2_ pair is bound to a ligand, or 3) both S_1_ and S_2_ sites in the pocket are bound to the same ligand (crosslinked). The same argument applies to the other binding pocket in the dimeric receptor. Using this representation, we grouped the individual states of four S_i_’s into states of two pairs of binding pockets.

3. S*caling*. After bunching IGF1 binding sites and decoupling tyrosine sites, the model accounts for six forms of receptor, with two identical tyrosine sites associated with each dimeric receptor form. Because the two sites in the same dimer form are assumed to act independently, one can construct a model as if there are six forms of receptor, each of which has only one tyrosine site. To preserve mass-action kinetics, one must increase the abundance of the ligand and receptor each by a factor of two and reduce the second-order rate constants characterizing interactions between the ligands and receptors (*a*_*1*_ and *a*_*2*_) by a factor of two.

Restructuration implies a model form that is equivalent to the natural formulation of a model, but with the elimination of many redundant states. As such, our restructured model is equivalent to the original model from which it was derived. We discuss this equivalency mathematically in S1 File, and through numerical simulation in S1 Fig.

### Parameter estimation

Parameter estimation was performed with the BioNetFit software package [61]. BioNetFit is a tool designed to fit BNGL-formatted models to data through use of an evolutionary algorithm. Evolutionary algorithms are population-based metaheuristics inspired by nature that involve independent evaluation of parameter sets across parameter space and perturbation of the parameters over multiple population generations. While fitting the IGF1R model to experimental data, we found and fixed a number of minor bugs in the BioNetFit code base. The modified source code for the version used in this work, v1.01, is available at https://github.com/RuleWorld/BioNetFit. To ensure that the initial set of parameter values effectively sample parameter space where distinct parameters may span multiple orders of magnitude, we normalize the parameters through the use of multiplicative factors so that each parameter can be perturbed on the same scale (see S2 Compressed/ZIP File Archive). Finally, we used a bootstrapping procedure that is embedded in BioNetFit to characterize the uncertainty of the fitted parameters. This procedure samples the experimental data points with replacement and refits the model to the bootstrap sample [104]. The 2.5% and 97.5% percentiles of observed parameters from 2,000 independent fits to bootstrap samples were used to create the 95% confidence interval. To obtain the confidence interval for parameter *a'*_*2*_, we calculated the value of *a'*_*2*_ using Eq 1 for each of the 2,000 bootstrapping fits, and calculated the confidence interval using percentiles in the same manner as for the other parameters.

### Formulation of cell line-specific models

The generic IGF1R signaling model can be explicitly parameterized as a cell line-specific model. To define a cell line-specific model, we incorporate signaling protein copy numbers consistent with the corresponding cell type from datasets available in the literature (S1 and S2 Tables). To make a model representing IGF1R signaling in a HeLa S3 cell, we specify abundances of the signaling proteins consistent with their reported copy numbers in HeLa S3 cells [63]. Hein et al. [69] provided absolute copy numbers for HeLa Kyoto cells. Deshmukh et al. [70] reported normalized relative abundances for C2C12 cells. Geiger et al. [67] quantified protein abundances in A549, GAMG, HEK293, HeLa S3, HEPG2, Jurkat, K562, LnCap, MCF7, RKO, and U2OS cell lines. Models for the NCI-60 cell lines were parameterized with data from Gholami et al. [68]. We used the higher coverage deep proteomics data for the seven cell lines for which these data were available: MCF7, M14, COLO205, CCRFCEM, U251, H460, PC3, SKOV3, and RXF393. Of the remaining NCI-60 cell lines, 24 had IGF1R expression that was nonzero. The proteomics profiling data were used to parameterize models for these cell lines. To calculate copy numbers from relative abundances, we assumed the same copy number of IGF1R as reported by Kulak et al. [63] and scaled the other copy numbers accordingly. This assumption is acceptable because we are interested in determining only the relative rank ordering of binding partner recruitment. With the exception of the protein copy numbers, the model assumes other parameters of the generic model remain the same across different cell types.

### Formulation of population-level models

We expanded the IGF1R signaling models into population-level models, which account for intrinsic and extrinsic contributions to cell-to-cell variability in expression of IGF1R and IGF1R-binding proteins across populations of clonal cells. In the population-level models, the copy number of any protein *i* in cell *k* is a value sampled from a log normal distribution: *X*_*k*,*i*_ = e^*μi+σiN*^. Here, *μ*_*i*_ is the mean and *σ*_*i*_ is the standard deviation. The mean *μ*_*i*_ represents the natural logarithm of the population-averaged copy number of the corresponding protein used in single-cell IGF1R signaling models. The population averaged copy numbers are listed in S1 Table. We assumed the same standard deviation *σ*_*i*_ = 0.2 for all proteins, a value consistent with experimentally determined cell-to-cell variability in protein copy number [105,106]. *N* is a random variable sampled from the standard normal distribution. Scripts used to generate the population models are provided in S3 Compressed/ZIP File Archive.

### Simulations

For the restructured model, BioNetGen was used to generate a system of ODEs. We numerically integrated the resulting system of equations using CVODE/SUNDIALS [107,108], which is included in the BioNetGen package. For the natural model formulation, because of the intractability of generating the large reaction network described by model rules, we performed network-free simulation using NFsim [35].

### Clustering

Recruitment data were obtained from steady-state simulations in individual cell line models. We normalized recruitment of each protein as the ratio of the absolute number of bound copies of the protein over the total amount of IGF1R. Some proteins can bind to IGF1R in multiple locations, so the ratio can be greater than one. Hierarchical clustering of cell lines and binding proteins according to normalized recruitment was performed with Python 2.7 and the clustermap function of Seaborn [109]. Clustering linkage was per Ward’s method [110].

### Ranking and evaluation of RTK-protein interactions

Ranks in Fig 4 and S2 File were determined using five different metrics, which are based on either 1) copy number information only, 2) *K*_*D*_ values only, 3) ratios of copy number to *K*_*D*_ values only, 4) the rule-based model simulation for single cells, which includes protein copy numbers, site-specific *K*_*D*_ values, and accounts for competition for phosphotyrosine sites, and 5) the analytical approximation, which includes copy number, *K*_*D*_ values and a simplified treatment of competition in which only one binding partner can interact with an IGF1R dimer at one time (S3 File).

To calculate correlations between the recruitment of signaling protein pairs, we considered a population of 5,000 cells for each of the cell line-specific population-level models. In the models, the cells were stimulated with 1 nM IGF1. We created the correlation matrices in Fig 7, S4 Fig and S5 Fig based on steady-state recruitment of proteins to IGF1R. For a given protein *i*∈{1, …, *m*}, where *m* = 18, we collected its steady-state IGF1R binding *Y*_*k*,*i*_ in cell *k*∈{1, …, *n*}, where *n =* 5,000. We calculated the mean receptor recruitment of the protein as $y_{i}=\sum_{k=1}^{n}y_{k,i}$ and the standard deviation follows as $\sigma_{i}=\sqrt{\left\{\sum_{k=1}^{n}{\left(Y_{k,i}-y_{i}\right)}^{2}/\left(n-1\right)\right\}}$.

Finally, the Pearson's correlation coefficient between each pair of proteins *i* and *j* is $p_{i,j}=cov\frac{\left(Y_{i},Y_{j}\right)}{\sigma_{i}\sigma_{j}}$, where $cov(Y_{i},Y_{j})=\sum_{k=1}^{n}\left(Y_{k,i}-y_{i}\right)(Y_{k,j}-y_{j})$. We used SciPy to calculate correlation coefficients from population-level data.

## Supporting information

S1 File A tutorial overview of model restructuration. Includes historical background and detailed discussion of decoupling, bunching, and scaling with examples.
(PDF)

S2 File Rank ordering of IGF1R binding partners in cell lines according to each of five metrics. Contains ranking tables for all cell lines evaluated in this study. The five metrics for ranking recruitment were 1) numerical simulations with the restructured model, 2) exact results from the analytical equilibrium binding model, 3) copy number, 4) *K*_*D*_, and 5) the ratio of copy number to *K*_*D*_.
(XLSX)

S3 File Derivation of the analytical approximation of equilibrium binding of SH2/PTB-containing proteins to an RTK. We derive analytical equations to predict binding of signaling proteins to an RTK and compare results to those of the numerical simulation described in the main text. Furthermore, we present a simplified set of equations, which provides a rule of thumb for estimating rank.
(PDF)

S1 Table Protein copy numbers in all cell lines. This table summarizes protein copy number values reported in the literature [63,68–70] and used in this study.
(XLSX)

S2 Table *K_D_* values for IGF1R binding partners. This table summarizes equilibrium dissociation constant values reported in the literature [45,46,89] and used in this study.
(XLSX)

S3 Table Summary of rank ordering of IGF1R binding partners across 45 cell line-specific models. (XLSX)

S1 Fig Exactness is maintained in the restructured formulation of the IGF1R signaling model. Plots show time courses of bound phosphotyrosine sites and bound signaling proteins from simulations of the HeLa S3 model in the natural formulation and the restructured formulation.
(TIFF)

S2 Fig Illustration of model restructuration. Cartoons of (A) bunching (B) decoupling, and (C-D) scaling are shown. (A) We can couple an S_1_ site from one IGF1R monomer and the S_2_ site from the other IGF1R monomer into one binding pocket, P. In the natural formulation, four different binding sites can be either free or bound to IGF1. In the restructured formulation, two binding pockets can each be free (white circle), bound to IGF1 (gray circle with IGF1), or crosslinked (black circle with IGF1). (B) We decouple each of the phosphotyrosine sites from the others, since the state of one site does not influence the state of any other site. In the restructured formulation, we consider six forms of the receptor, each with only one possible tyrosine residue. (C) Each phosphotyrosine residue can be either dephosphorylated, phosphorylated and free (green circle), or phosphorylated and bound (green circle plus yellow pentagon). If we consider receptor monomers instead of dimers, the minimum number of possible states is reduced from six to three. (D) Upon the above restructuring, to preserve mass-action kinetics, the rate constant for ligand binding must be halved and the total receptor and ligand concentrations must be doubled.
(PDF)

S3 Fig Comparison of quantitative predictions from numerical simulations and the analytical approximation for HeLa S3 and HeLa Kyoto cell lines. Plots show the number of molecules of each protein bound at steady state predicted by either numerical simulations (x-axis) or the analytical approximation (y-axis). A dashed gray line on the diagonal illustrates perfect agreement. The Pearson’s correlation coefficient and *p* value are displayed for each dataset (calculated using R software’s cor.test).
(TIFF)

S4 Fig Pairwise correlations for IGF1R signaling protein recruitment in lung, colon, renal, liver, melanoma, leukemia, and mouse cell lines. Red indicates a negative Pearson’s *r*, blue indicates a positive Pearson’s *r*, and white indicates no correlation between each pair of proteins.
(TIFF)

S5 Fig Pairwise correlations for IGF1R signaling protein recruitment in breast, cervical, prostate, central nervous system, and bone cell lines. Red indicates a negative Pearson’s *r*, blue indicates a positive Pearson’s *r*, and white indicates no correlation between each pair of proteins.
(TIFF)

S1 Compressed File Archive Cell-specific IGF1R signaling models. A compressed archive containing the files for 45 cell line-specific models. Both natural and restructured models are included for HeLa S3. Only the restructured models are included for the other cell lines. BNGL models are plain-text files with the filename extension .bngl, intended for processing by BioNetGen.
(ZIP)

S2 Compressed File Archive Parameter estimation files with BioNetFit. This compressed directory contains the plain-text files that were processed by BioNetFit to estimate the parameters given in Table 1. The configuration file (filename extension .conf) provides instructions for parameter estimation. The .bngl file contains the IGF1-IGF1R binding model. The .exp files contain experimental data from Fig 5B and 5D in Kiselyov et al. [44].
(ZIP)

S3 Compressed File Archive Population models. This compressed directory contains the files and scripts needed to generate models for a clonal population of cells with variability in protein copy number. The Python script “population_model.py” generates BioNetGen input files. These files can then be processed by BioNetGen to generate steady-state recruitment data for all cells in a population. The script “pearson_calc.py” calculates the correlations between pairs of proteins, taking the results of simulations as input. The file “bngl_pop_editor.py” contains functions called in the executable files.
(ZIP)
