# Supplementary material for: Modeling cell line-specific recruitment of signaling proteins to the insulin-like growth factor 1 receptor
Source: PLoS Comput Biol. 2019 Jan 17;15(1):e1006706. doi: 10.1371/journal.pcbi.1006706 (PMC6353226; doi:10.1371/journal.pcbi.1006706)
Supplement: S1 File — Includes historical background and detailed discussion of decoupling, bunching, and scaling with examples. (PDF) [file pcbi.1006706.s001.pdf]

A Tutorial Overview of Model Restructuration

This document was prepared as Supporting Information (S1 File) for "Modeling cell line-specific recruitment of signaling proteins to the insulin-like growth factor 1 receptor."

Keesha E. Erickson[1], Oleksii S. Rukhlenko[2], Md Shahinuzzaman[3], Kalina P. Slavkova[1,4], Yen Ting Lin[1,4], Ryan Suderman[1,4], Edward C. Stites[5], Marian Anghel[6], Richard G. Posner[7], Dipak Barua[3], Boris N. Kholodenko[2,8], and William S. Hlavacek[1,4*]

[1]Theoretical Biology and Biophysics Group, Theoretical Division, Los Alamos National Laboratory, Los Alamos, New Mexico, USA. [2]Systems Biology Ireland, University College Dublin, Belfield, Dublin, Ireland. [3]Department of Chemical and Biochemical Engineering, University of Missouri Science and Technology, Rolla, Missouri, USA. [4]Center for Nonlinear Studies, Los Alamos National Laboratory, Los Alamos, New Mexico, USA. [5]The Salk Institute for Biological Studies, La Jolla, California, USA. [6]Information Sciences Group, Computer, Computational and Statistical Sciences Division, Los Alamos National Laboratory, Los Alamos, New Mexico, USA. [7]Department of Biological Sciences, Northern Arizona University, Flagstaff, Arizona, USA. [8]School of Medicine and Medical Science and Conway Institute of Biomolecular and Biomedical Research, University College Dublin, Belfield, Dublin, Ireland.

[*]Corresponding author. Email: wish@lanl.gov

1 Introduction and historical background

Rule-based models, which comprise parameters, molecule type definitions (also called agents), and rules, may sometimes be profitably transformed via a process that we call here "restructuration." The process is known by various names in the literature (and has sometimes been used without reference to any special name for it). Restructuration is useful, but it is not commonly used—there are only a handful of examples of its profitable application in modeling studies—and it is sometimes confused with model reduction. For these reasons and as a complement to the Materials and Methods section of the main text, we provide this tutorial overview of restructuration.

Model restructuration is distinct from model reduction or coarse graining. In fact, restructuration is analogous to rewriting a differential equation in dimensionless form, which is clearly not reduction. In our view, a model reduction entails a loss of information, i.e., the introduction of some sort of approximation for the purpose of obtaining a simpler model form. An example of model reduction in the context of rule-based modeling is provided by the study of Faeder et al. (2005). In this study, variables and the corresponding equations/terms were systematically removed from a rule-derived system of coupled ordinary differential equations (ODEs) to obtain a simpler model form that still makes predictions largely consistent with the original model. Other examples are provided by the studies of Klinke and Finley (2012) and Birtwistle (2015). In contrast, a restructured model is entirely equivalent (in terms of information about system state) to the original model from which it is derived. One could consider a restructuring to be an "exact reduction," but this term is potentially confusing. The reason "reduction" is used in the literature is because, after profitable restructuring, a transformed model will imply a smaller reaction network (and shorter list of corresponding ODEs) than the rules of the original model formulation. One should be careful not to confuse a rule-based model with the reaction network implied by the model. The main purpose of deriving a reaction network from the rules of a model is to implement an indirect simulation method (Chylek et al., 2014), such as numerical integration of the corresponding ODEs. (An indirect simulation method is one that depends on transformation of a rule-based model into a conventional form, such as that of ODEs.) For naturally formulated rule-based models, the variables of the ODEs obtained through network generation tend to redundantly characterize system state, as we will see later through examples. (It should be noted that we are not referring here to an established technical definition of "redundancy," as in, for example, linear algebra or information theory.) The goal of model restructuration is to reduce redundancy without loss of information about system state. Importantly, for a given rule-based model, we want to find a restructuring of the model as given (i.e., a transformation of the model's parameters, molecule type definitions, and/or rules), not of the reaction network or equations implied by the model. This is because restructuring is most useful when network generation is impracticable, i.e., when deriving the reaction network and corresponding ODEs implied by a rule-based model is not feasible.

Before moving on, let us briefly introduce the model elements that are potentially affected by restructuration. **Parameters** are constant quantities such as empirical rate constants and initial/total abundances of molecules/chemical species. **Molecule type definitions** provide formal representations of the types of molecules considered in a model, each of which may have multiple sites, which in turn may have multiple internal states and interaction partners. The allowed states and interactions are defined jointly by molecule type definitions and rules. **Rules** provide formal representations of molecular interactions, transformations/modifications of (bio)molecules (e.g., protein phosphorylation), and other processes (e.g., synthesis, degradation, and transport). Several recent reviews of rule-based modeling are available (Chylek et al., 2013; 2014; Stefan et al., 2014).

As noted earlier, a natural formulation of a rule-based model (i.e., a straightforward formaliza-

tion of mechanistic knowledge) tends to imply a reaction-network representation of the system of interest in which the state variables (the concentrations of all chemical species in the network) are redundant. In other words, the system state can be fully defined by a number of variables that tends to be (much) less than the number of conventional state variables (the concentrations of the potentially populated chemical species). By restructuring a rule-based model, this redundancy can be reduced, sometimes dramatically.

Here, we discuss three particular restructuring strategies for reducing redundancy, which we term 1) decoupling of independent sites, or simply **decoupling**; 2) "bunching" to obtain implicit and concise representation of assemblies (e.g., using features of a single molecule type definition to represent features of connected molecules), or simply **bunching**; and 3) site degeneracy elimination with invariant scaling, or simply **scaling**. There are other strategies (Petrov et al., 2012; Feret et al., 2012; 2013; Bugenhagen and Beard, 2012) and there may be additional strategies that are yet to be discovered and demonstrated. The first two strategies have been considered in various earlier studies. The vast majority of these studies are methodological but they include very early applications of the rule-based modeling approach in systems biology. For example, implicit representation of complexes (bunching) was used by Faeder et al. (2003) to formulate a model for IgE receptor signaling, and site decoupling was used by Blinov et al. (2006) to formulate a rule-based model for epidermal growth factor receptor (EGFR) signaling based on the interactions considered in the earlier ODE model of Kholodenko et al. (1999). The benefits of site decoupling in the context of biological rule-based modeling were perhaps first discussed in depth by Borisov et al. (2005; 2006), and automated restructuration methodology was perhaps first implemented in software (as a component of BioNetGen, which is now deprecated) by Borisov et al. (2008). The work of Gilles and co-workers is also notable (Conzelmann et al., 2004; 2008; Koschorreck et al., 2007).

In our opinion, the best nomenclature for restructuration methods remains open for discussion. We note that the decoupling strategy discussed here corresponds to the rigorously defined and formally described transformation that has been called "fragmentation" by Feret et al. (2009). Feret et al. (2009) provide a systematic algorithm for fragmentation. The steps of this recipe are easy to perform manually. Feret et al. (2009) also introduced the term "compression" for essentially what we call bunching. We do not have strong objections to the nomenclature of Feret et al. (2009), but we feel that the alternative terms used here better connote the essence of the corresponding transformations. We do feel that it is important to distinguish these methods from methods for model reduction and coarse graining, which motivates our use of the term "restructuration," which has the following apropos dictionary definition: "The action of giving a new structure to something" (*Oxford Dictionaries*, https://en.oxforddictionaries.com/definition/restructuration, Accessed 28 September 2018).

Our goal is not to present a comprehensive survey of the "restructuration" literature. Most reports about restructuration can likely be found by looking for papers that cite the early studies referenced above. We also do not intend to claim that our ideas about restructuration are entirely novel. Of the three restructuration strategies that we discuss here, only the scaling strategy is novel, to our best knowledge. The main novelty is the *application* of restructuration to our detailed model for early insulin-like growth factor 1 (IGF1) receptor (IGF1R) signaling events. A related restructuration has been presented by Feret et al. (2009) for a model for EGFR signaling. The study of Birtwistle (2015) is also related. This study, which involved use of a model for EGFR signaling for demonstration purposes, presents methodology that entails actual model reduction, in that the methodology leverages quasi-equilibrium assumptions.

The three restructuring strategies discussed here are likely to be broadly applicable but how exactly they are applied (or even if they are applicable) will vary from problem to problem. The goal of site decoupling is to represent independent molecular sites independently. When the state

of a site within a molecule changes in a manner that is independent of the state of other sites in the same molecule, independent representation of this site can sometimes be beneficial. The goal of bunching is to simplify the representation of molecular assemblies. Instead of tracking the composition and connectivity of an assembly explicitly, an assembly is represented in terms of the features of a single molecule type definition. Finally, site degeneracy elimination with invariant scaling offers a means for replacing multiple equivalent sites with a single site by compensating for the reduced degeneracy, κ sites $\rightarrow$ 1 site (where the integer κ is greater than 1), through appropriate scaling of relevant model parameter values.

Each of the three restructuring strategies is illustrated below through simple examples. As we will see clearly, in these examples, restructuration does not involve any approximations or loss of information. The benefit of restructuration is a rule-implied reaction network that is smaller than that implied by the original model formulation, when measured in terms of the number of potentially populated chemical species or the number of reactions connecting these species. The cost is a less natural, less readable model formulation. Interestingly, contrary to the notion of "reduction," a restructured model formulation is usually (if not always) more verbose than the original natural formulation.

Importantly, the restructuration strategies discussed here are only applicable when rules are translated into ODEs. These strategies should not be used when stochastic simulations are of interest. Methods for stochastic models are distinct and they are discussed elsewhere (Feret et al., 2013). Software (Complx) is available (https://github.com/kappamodeler/kappa). When interested in stochastic behavior, in lieu of restructuration, one could simply apply a network-free simulation method (Danos et al., 2007; Yang et al., 2008; Colvin et al., 2009; 2010; Yang and Hlavacek, 2011; Sneddon et al., 2011; Boutillier et al., 2017; 2018; Lin et al., 2018). Such methods, which are related to Gillespie's stochastic simulation algorithm (Gillespie, 2007), are kinetic Monte Carlo procedures that do not require the generation of a reaction network (Suderman et al., 2018). In network-free methods, the rules of a model serve as reaction event generators. For this reason, network-free methods are also called direct methods. The dichotomy of direct and indirect methods is discussed by Chylek et al. (2014).

2 Decoupling the representation of sites within a molecule when these sites behave independently

The most straightforward model restructuration strategy is that of site decoupling. The goal of decoupling is to represent independent sites independently.

Consider a receptor R with three sites S^X, S^Y and S^Z having states x, y, and z, respectively. We will assume that each site may be found in only one of two internal states (0 or 1). Thus, there are 8 possible combinations of site states (and equivalently, 8 possible receptor states):

$$(x, y, z) \in [(0,0,0), (1,0,0), (0,1,0), (0,0,1), (1,1,0), (1,0,1), (0,1,1), (1,1,1)]$$

We will take any transition between site states to occur spontaneously in accordance with a first-order rate law. Thus, transitions between the 8 possible receptor states are described by the following list of reactions:

$$R_{000} \xrightarrow{k_{+1}} R_{100} \quad (1)$$

$$R_{100} \xrightarrow{k_{-1}} R_{000} \quad (2)$$

$$R_{000} \xrightarrow{k_{+2}} R_{010} \quad (3)$$

$$R_{010} \xrightarrow{k_{-2}} R_{000} \tag{4}$$

$$R_{000} \xrightarrow{k_{+3}} R_{001} \tag{5}$$

$$R_{001} \xrightarrow{k_{-3}} R_{000} \tag{6}$$

$$R_{100} \xrightarrow{k_{+4}} R_{110} \tag{7}$$

$$R_{110} \xrightarrow{k_{-4}} R_{100} \tag{8}$$

$$R_{100} \xrightarrow{k_{+5}} R_{101} \tag{9}$$

$$R_{101} \xrightarrow{k_{-5}} R_{100} \tag{10}$$

$$R_{010} \xrightarrow{k_{+6}} R_{110} \tag{11}$$

$$R_{110} \xrightarrow{k_{-6}} R_{010} \tag{12}$$

$$R_{010} \xrightarrow{k_{+7}} R_{011} \tag{13}$$

$$R_{011} \xrightarrow{k_{-7}} R_{010} \tag{14}$$

$$R_{001} \xrightarrow{k_{+8}} R_{101} \tag{15}$$

$$R_{101} \xrightarrow{k_{-8}} R_{001} \tag{16}$$

$$R_{001} \xrightarrow{k_{+9}} R_{011} \tag{17}$$

$$R_{011} \xrightarrow{k_{-9}} R_{001} \tag{18}$$

$$R_{110} \xrightarrow{k_{+10}} R_{111} \tag{19}$$

$$R_{111} \xrightarrow{k_{-10}} R_{110} \tag{20}$$

$$R_{101} \xrightarrow{k_{+11}} R_{111} \tag{21}$$

$$R_{111} \xrightarrow{k_{-11}} R_{101} \tag{22}$$

$$R_{011} \xrightarrow{k_{+12}} R_{111} \tag{23}$$

$$R_{111} \xrightarrow{k_{-12}} R_{011} \tag{24}$$

where R_{xyz} is the receptor state corresponding to the (x, y, z) combination of site states and the k terms are rate constants. The well-mixed mass-action kinetics of this reaction network are described (in the continuum limit) by the following system of coupled ODEs:

$$\frac{d}{dt}\begin{pmatrix} R_{000} \\ R_{100} \\ R_{010} \\ R_{001} \\ R_{110} \\ R_{101} \\ R_{011} \\ R_{111} \end{pmatrix} = \begin{pmatrix} -\delta_1 & k_{-1} & k_{-2} & k_{-3} & 0 & 0 & 0 & 0 \\ k_{+1} & -\delta_2 & 0 & 0 & k_{-4} & k_{-5} & 0 & 0 \\ k_{+2} & 0 & -\delta_3 & 0 & k_{-6} & 0 & k_{-7} & 0 \\ k_{+3} & 0 & 0 & -\delta_4 & 0 & k_{-8} & k_{-9} & 0 \\ 0 & k_{+4} & k_{+6} & 0 & -\delta_5 & 0 & 0 & k_{-10} \\ 0 & k_{+5} & 0 & k_{+8} & 0 & -\delta_6 & 0 & k_{-11} \\ 0 & 0 & k_{+7} & k_{+9} & 0 & 0 & -\delta_7 & k_{-12} \\ 0 & 0 & 0 & 0 & k_{+10} & k_{+11} & k_{+12} & -\delta_8 \end{pmatrix} \begin{pmatrix} R_{000} \\ R_{100} \\ R_{010} \\ R_{001} \\ R_{110} \\ R_{101} \\ R_{011} \\ R_{111} \end{pmatrix} \tag{25}$$

where the R terms now represent concentrations. The diagonal terms in the matrix above are defined as follows:

$$\delta_1 \equiv k_{+1} + k_{+2} + k_{+3} \tag{26}$$

$$\delta_2 \equiv k_{-1} + k_{+4} + k_{+5} \quad (27)$$
$$\delta_3 \equiv k_{-2} + k_{+6} + k_{+7} \quad (28)$$
$$\delta_4 \equiv k_{-3} + k_{+8} + k_{+9} \quad (29)$$
$$\delta_5 \equiv k_{-4} + k_{-6} + k_{+10} \quad (30)$$
$$\delta_6 \equiv k_{-5} + k_{-8} + k_{+11} \quad (31)$$
$$\delta_7 \equiv k_{-7} + k_{-9} + k_{+12} \quad (32)$$
$$\delta_8 \equiv k_{-10} + k_{-11} + k_{-12} \quad (33)$$

Let us use S_0^X, S_1^X, S_0^Y, S_1^Y, S_0^Z, and S_1^Z to denote the concentrations of the three sites in each of their two allowed states. These concentrations of site forms are related to the concentrations of receptor forms as follows:

$$S_0^X \equiv R_{000} + R_{010} + R_{001} + R_{011} \quad (34)$$
$$S_1^X \equiv R_{100} + R_{110} + R_{101} + R_{111} \quad (35)$$
$$S_0^Y \equiv R_{000} + R_{100} + R_{001} + R_{101} \quad (36)$$
$$S_1^Y \equiv R_{010} + R_{110} + R_{011} + R_{111} \quad (37)$$
$$S_0^Z \equiv R_{000} + R_{100} + R_{010} + R_{110} \quad (38)$$
$$S_1^Z \equiv R_{001} + R_{101} + R_{011} + R_{111} \quad (39)$$

From Equations (1)–(24) and (34)–(39), we can find expressions for the time derivatives of the S variables (in terms of the R variables). For example, we find

$$\frac{dS_0^X}{dt} = -(k_{+1}R_{000} + k_{+6}R_{010} + k_{+8}R_{001} + k_{+12}R_{011}) \quad (40)$$
$$+(k_{-1}R_{100} + k_{-6}R_{110} + k_{-8}R_{101} + k_{-12}R_{111}) \quad (41)$$

and

$$\frac{dS_1^X}{dt} = (k_{+1}R_{000} + k_{+6}R_{010} + k_{+8}R_{001} + k_{+12}R_{011}) \quad (42)$$
$$-(k_{-1}R_{100} + k_{-6}R_{110} + k_{-8}R_{101} + k_{-12}R_{111}) \quad (43)$$

If transitions between states 0 and 1 of site S^X occur independently of the states of the other sites in R (a prerequisite for restructuring via site decoupling), then $k_{+1} = k_{+6} = k_{+8} = k_{+12}$ and $k_{-1} = k_{-6} = k_{-8} = k_{-12}$. If the same also holds for sites S^Y and S^Z, we obtain the following equations:

$$-dS_0^X/dt = dS_1^X/dt = k_+^X S_0^X - k_-^X S_1^X \quad (44)$$
$$-dS_0^Y/dt = dS_1^Y/dt = k_+^Y S_0^Y - k_-^Y S_1^Y \quad (45)$$
$$-dS_0^Z/dt = dS_1^Z/dt = k_+^Z S_0^Z - k_-^Z S_1^Z \quad (46)$$

where

$$k_+^X \equiv k_{+1} = k_{+6} = k_{+8} = k_{+12} \quad (47)$$
$$k_-^X \equiv k_{-1} = k_{-6} = k_{-8} = k_{-12} \quad (48)$$
$$k_+^Y \equiv k_{+2} = k_{+4} = k_{+9} = k_{+11} \quad (49)$$
$$k_-^Y \equiv k_{-2} = k_{-4} = k_{-9} = k_{-11} \quad (50)$$

$$k_+^Z \equiv k_{+3} = k_{+5} = k_{+7} = k_{+10} \tag{51}$$

$$k_-^Z \equiv k_{-3} = k_{-5} = k_{-7} = k_{-10} \tag{52}$$

We note that Equations (44)–(46) are the chemical kinetics equations that we would have written if we had started with the following list of reactions:

$$S_0^X \xrightarrow{k_+^X} S_1^X \tag{53}$$

$$S_1^X \xrightarrow{k_-^X} S_0^X \tag{54}$$

$$S_0^Y \xrightarrow{k_+^Y} S_1^Y \tag{55}$$

$$S_1^Y \xrightarrow{k_-^Y} S_0^Y \tag{56}$$

$$S_0^Z \xrightarrow{k_+^Z} S_1^Z \tag{57}$$

$$S_1^Z \xrightarrow{k_-^Z} S_0^Z \tag{58}$$

Thus, because of the assumptions made about the independence of receptor sites, there are two equivalent mathematical descriptions of the same system: Equation (25) and Equations (44)–(46). These sets of equations correspond to different lists of reactions: Equations (1)–(24) and Equations (53)–(58).

Likewise, there are two equivalent rule-based model formulations for the system of interest, which are given in Figures A and B. One can easily confirm—by using BioNetGen (Blinov et al., 2004; Harris et al., 2016) to generate the reaction networks implied by the two models—that the models of Figures A and B imply the reaction networks defined by Equations (1)–(24) and Equations (53)–(58), respectively. The main difference between the two rule-based model formulations is that in the model formulation of Figure A the receptor is represented as having three sites. In contrast, in the model formulation of Figure B, each receptor site is represented independently. Figure A presents the natural model formulation, and Figure B presents the restructured model formulation.

At this point, we should call attention to a peculiar feature of rule-based models, or least rule-based models for biomolecular site dynamics. These models tend to account for a large number of chemical species, because interactions among multi-site, multi-state biomolecules tend have the potential to generate numerous species. This feature of biomolecular interaction networks has been called combinatorial complexity (Hlavacek et al., 2003). Thus, when one creates a BioNetGen input file to define a rule-based model, the file typically also includes definitions of the specific simulation outputs of interest. Within the framework of the BioNetGen language (BNGL) (Faeder et al., 2009; Hogg et al., 2014), simulation outputs are defined as observables and global functions. An observable is a sum of the abundances of species or moieties matched by a user-defined pattern. A global function is a user-defined mathematical function of observables and/or parameters. Usually one is interested in only certain functions/readouts of system state, not the entire set of state variables, which would be an overwhelming amount of information. This is especially true because experimental readouts tend to reflect ensemble quantities, such as the overall level of phosphorylation of a particular site regardless of how that site is distributed amongst the populated chemical species within a cellular network. With the natural formulation of Figure A, outputs corresponding to concentrations of receptor phosphoforms can be defined in a straightforward manner, because all possible receptor phosphoforms are included explicitly in the rule-derived reaction network. With the restructured model formulation of Figure B, the exact same outputs can be defined, but their

definitions are more complex. The BNGL files of Figures A and B both request that BioNetGen report the concentration of receptors having all three receptor sites in state 1 (cf. the `R111` observable output defined in Figure A and the `R111()` global function output defined in Figure B). This illustrates that both models fully account for system state; there is no loss of state information in transforming the model formulation of Figure A into the model formulation of Figure B. We elaborate on this point below.

In summary, in the model of Figure A, which corresponds to the list of reactions given by Equations (1)–(24), we have assigned each rate constant a value of 1 (per unit time). Thus, the mass-action chemical kinetics of the system represented by this model are characterized by the following system of coupled ODEs, which is a special case of Equation (25):

$$\frac{d}{dt}\begin{pmatrix} R_{000} \\ R_{100} \\ R_{010} \\ R_{001} \\ R_{110} \\ R_{101} \\ R_{011} \\ R_{111} \end{pmatrix} = \begin{pmatrix} -3 & 1 & 1 & 1 & 0 & 0 & 0 & 0 \\ 1 & -3 & 0 & 0 & 1 & 1 & 0 & 0 \\ 1 & 0 & -3 & 0 & 1 & 0 & 1 & 0 \\ 1 & 0 & 0 & -3 & 0 & 1 & 1 & 0 \\ 0 & 1 & 1 & 0 & -3 & 0 & 0 & 1 \\ 0 & 1 & 0 & 1 & 0 & -3 & 0 & 1 \\ 0 & 0 & 1 & 1 & 0 & 0 & -3 & 1 \\ 0 & 0 & 0 & 0 & 1 & 1 & 1 & -3 \end{pmatrix} \begin{pmatrix} R_{000} \\ R_{100} \\ R_{010} \\ R_{001} \\ R_{110} \\ R_{101} \\ R_{011} \\ R_{111} \end{pmatrix} \tag{59}$$

The rank of the matrix in Equation (59) is 7, which reflects conservation of receptor (i.e., 1 invariant quantity). Because site state transitions occur independently, meaning that Equations (47)–(52) are satisified, Equation (59) can be rewritten in terms of new variables, which correspond to the state variables of the restructured model of Figure B, as follows:

$$\frac{d}{dt}\begin{pmatrix} S_0^X \\ S_1^X \\ S_0^Y \\ S_1^Y \\ S_0^Z \\ S_1^Z \end{pmatrix} = \begin{pmatrix} -1 & 1 & 0 & 0 & 0 & 0 \\ 1 & -1 & 0 & 0 & 0 & 0 \\ 0 & 0 & -1 & 1 & 0 & 0 \\ 0 & 0 & 1 & -1 & 0 & 0 \\ 0 & 0 & 0 & 0 & -1 & 1 \\ 0 & 0 & 0 & 0 & 1 & -1 \end{pmatrix} \begin{pmatrix} S_0^X \\ S_1^X \\ S_0^Y \\ S_1^Y \\ S_0^Z \\ S_1^Z \end{pmatrix} \tag{60}$$

where

$$\begin{pmatrix} S_0^X \\ S_1^X \\ S_0^Y \\ S_1^Y \\ S_0^Z \\ S_1^Z \end{pmatrix} = \begin{pmatrix} 1 & 0 & 1 & 1 & 0 & 0 & 1 & 0 \\ 0 & 1 & 0 & 0 & 1 & 1 & 0 & 1 \\ 1 & 1 & 0 & 1 & 0 & 1 & 0 & 0 \\ 0 & 0 & 1 & 0 & 1 & 0 & 1 & 1 \\ 1 & 1 & 1 & 0 & 1 & 0 & 0 & 0 \\ 0 & 0 & 0 & 1 & 0 & 1 & 1 & 1 \end{pmatrix} \begin{pmatrix} R_{000} \\ R_{100} \\ R_{010} \\ R_{001} \\ R_{110} \\ R_{101} \\ R_{011} \\ R_{111} \end{pmatrix} \tag{61}$$

One can easily confirm that Equation (60) follows from Equation (59) and the change of variables defined by Equation (61). It happens to be the case that the change of variables defined by Equation (61) produces a self-consistent set of equations, Equation (60). This is not generally the case for an arbitrary change of variables. The rank of the matrix in Equation (60) is 3, which reflects conservation of sites S^X, S^Y, and S^Z (i.e., 3 invariant quantities). From a probabilistic argument, $\mathrm{P}(x,y,z) = \mathrm{P}(x) \cdot \mathrm{P}(y) \cdot \mathrm{P}(z)$, we conclude that the original R variables are related to the new S variables as follows:

$$\frac{R_{ijk}}{R_T} = \left(\frac{S_i^X}{R_T}\right)\left(\frac{S_j^Y}{R_T}\right)\left(\frac{S_k^Z}{R_T}\right) \quad \text{for } i,j,k \in \{0,1\} \tag{62}$$

where

$$R_T = \sum_{i=0}^{1}\sum_{j=0}^{1}\sum_{k=0}^{1} R_{ijk} = \sum_{i=0}^{1} S_i^X = \sum_{j=0}^{1} S_j^Y = \sum_{k=0}^{1} S_k^Z \tag{63}$$

One can check that these relations are correct by substituting the expression given above for each R_{ijk} into Equation (61) and confirming that the equality in Equation (61) holds. Thus, the original model (Figure A) and the restructured model (Figure B) both fully account for system state, and restructuration does not entail a loss of information. The abundances of receptor states are not explicitly tracked in the restructured model as in the original model; however, these quantities are functions of the abundances of the site states (Equation (62)), which *are* tracked in the restructured model. We note that methods and software implementations for finding equivalent ODE models, as above, are available (Cardelli et al., 2017a; 2017b).

We have discussed restructuration as a transformation of a model, because one would typically start with a natural formulation of mechanistic knowledge. (Representation of a receptor with three sites of interest is naturally represented by a molecule type definition that accounts for all three sites.) However, an experienced modeler could perhaps bypass the step of creating a natural formulation and start immediately with a model form that implies conventional state variables having minimal redundancy.

3 Bunching to obtain an implicit and concise representation of assemblies

The second restructuring strategy entails a type of transformation that is difficult to explain succinctly. Thus, a jargon term would be useful. We propose "bunching," which is more singular than the term "compression" introduced by Feret et al. (2009). The goal of bunching is to replace explicit representation of the composition and connectivity of a molecular assembly with a simpler representation, ideally one involving use of only a single molecule type definition. For example, bunching may involve combining the representation of a molecule together with that of one or more of its direct/indirect binding partners through the addition of internal states to the molecule's formal molecule type definition. This restructuring strategy is best explained through examples. Here, we will consider an example that is distinct from that discussed in the main text. It is more or less the same example considered by Feret et al. (2009), which was inspired by the model of Blinov et al. (2006).

Let us consider a receptor tyrosine kinase (RTK) that is monomeric in the absence of a ligand and that is activated by ligand-stimulated dimerization. Autophosphorylation of tyrosines in the receptor is taken to depend on receptor dimerization. The receptor dimerizes via a receptor-receptor interface, and dimerization is promoted by ligand-receptor binding. The reason dimerization is promoted by ligand binding is because the ligand binds in a way that stabilizes a conformation of the receptor's ectodomain that is competent for receptor-receptor interaction. The specific RTK that we have in mind is EGFR (Burgess et al., 2003).

Because we assume that receptor autophosphorylation depends on receptor dimerization, we need to keep track of whether receptors are monomeric or dimeric to model autophosphorylation correctly. There are two ways to accomplish this task. A natural way is to represent receptor dimers as a connected pair of receptor monomers, as illustrated in Figure C or as in the model of Blinov et al. (2006). A drawback of this approach is the large number of distinct phosphoforms that are possible for receptor dimers. In the model of Figure C, a receptor monomer is taken to have five sites of autophosphorylation. Thus, a receptor monomer can potentially be found in any of $n = 2^5 = 32$

phosphoforms. A receptor dimer has a significantly larger number of possible phosphoforms, which is determined by combinatorics. In particular, a dimer has $n = 32$ symmetric phosphoforms and $n(n-1)/2 = 496$ asymmetric phosphoforms, for a total of 528 distinct phosphoforms.

A second way to keep track of whether receptors are monomeric or dimeric is to simply track the bound state of the part of the receptor that is responsible for dimerization (Figure D), which is called "`II`" in the models of Figures C and D, in reference to subdomain II of the EGFR ectodomain, which contains the so-called dimerization arm (Burgess et al., 2003). If this site is marked as free, the receptor is monomeric and cannot undergo autophosphorylation. If this site is marked as bound, the receptor is in a dimer and eligible to undergo autophosphorylation. The model of Figure D illustrates this approach for keeping track of receptor dimers; it is a restructured formulation of the model of Figure C obtained through bunching. In the model formulation of Figure D, the representation of receptor dimers is bunched together with the representation of receptor monomers in the molecule type definition for the receptor.

Before ending our discussion of bunching, we should call attention to a few features of the way that receptor dimer dissociation is represented in the restructured model of Figure D. Firstly, it should be noted that the rules that characterize dimer dissociation in the natural and restructured models of Figures C and D introduce equivalent first-order rate laws with rate constant `kmx` for dimer dissociation, which may not be immediately obvious. In the natural model, the dissociation rule (the reverse reading of the second rule in the `reaction rules` block, which is a reversible rule) acts on connected pairs of receptor monomers, and the product of rule application is two, unconnected receptor monomers. In the restructured model, the dissociation rule (the third rule in the `reaction rules` block, which is a unidirectional rule) acts on individual receptors marked as being in dimers, and the product of rule application is a single receptor monomer. Because receptors in dimers in the restructured model will always have twice the concentration of receptor dimers in the natural model, the rates of monomer formation via dimer dissociation in the two models will always be identical. Secondly, it should be noted that the dimer dissociation rules of Figures C and D provide an example of why reversible rules should be used with caution. (In a reversible rule, the symbol `<->` is used to separate the left-hand and right-hand sides of the rule, whereas the symbol `->` is used for this purpose in a unidirectional rule.) In the natural model, dimer formation and dissociation are represented compactly using a reversible rule, whereas in the restructured model, dimer formation and dissociation are represented by separate unidirectional rules. Separate unidirectional rules must used in the restructured model because (inappropriate) use of a reversible rule of the form

```
EGFR(I_III!+,II~m)+EGFR(I_III!+,II~m)<->EGFR(I_III!+,II~d)+EGFR(I_III!+,II~d) kpx,kmx
```

would make dimer dissociation a process having a second-order rate law, with an erroneous dependence of reaction rate on the square of the concentration of receptors in dimers. Thirdly, it should be noted that the representation of dimer dissociation in the restructured model depends on a continuum, mean-field description of chemical kinetics (i.e., use of numerical integration of ODEs as the simulation method). This rule would be inappropriate for representation of chemical kinetics at the resolution of discrete reaction events. In other words, the rule is incompatible with the use of methods such as Gillespie's stochastic simulation algorithm, because an individual dissociation reaction event consumes *two* receptors in dimers and concomitantly generates *two* receptor monomers, not just one. The rule is acceptable here because ODEs do not represent reaction kinetics at the level of discrete reaction events and the rule provides a correct mean-field representation of dissociation kinetics.

4 Site degeneracy elimination with invariant scaling

The goal of scaling is to eliminate site degeneracy, as illustrated in Figure E. The scenario of Figure E is one that arose in restructuring of our IGF1R signaling model. Before scaling, we are considering an IGF1R dimer with two copies of Y973, which is natural. In the physical world, IGF1R is constitutively dimeric and there is one copy of the tyrosine at position 973 in each monomeric subunit of a dimer. After scaling, dimers are represented as having only a single copy of Y973. This elimination of site degeneracy is accompanied by a scaling of parameter values. The total abundances of the ligand (IGF1) and the receptor dimer, $L_T^{\rm Y973}$ and $R_T^{\rm Y973}$, are each *increased* by a factor of 2, and the rate constants for ligand binding to sites S1 and S2 in the receptor dimer, which we denote here as k_{+1} and k_{+2}, are each *decreased* by a factor of 2. System dynamics are unaffected by this change in the way that a receptor dimer is represented. To see that this claim is correct, let us first note that the cytoplasmic abundance of Y973 is unchanged by scaling. The total abundance of Y973 is $2R_T^{\rm Y973}$ both before and after the scaling transformation. Moreover, let us recall that the processes of auotphosphorylation and dephosphorylation of any receptor site are modeled as first-order reactions and that each site is taken to behave independently of all other receptor sites (including its twin). Thus, the crux of the matter is the equivalence of ligand-receptor binding dynamics in the two model formulations.

To see that ligand-receptor binding dynamics are unaffected by scaling, let us consider the detailed reaction scheme for ligand-receptor binding shown in Figure F. ODEs for the mass-action kinetics of this reaction network can be written as follows:

$$\frac{dR_{00}}{dt} = -2k_{+1}LR_{00} + k_{-1}R_{10} - 2k_{+2}LR_{00} + k_{-2}R_{20} \tag{64}$$

$$\frac{dR_{10}}{dt} = 2k_{+1}LR_{00} - k_{-1}R_{10} - k_{+2}LR_{10} + k_{-2}R_{12} - k_{+1}LR_{10} + 2k_{-1}R_{11} - \lambda_{+2}R_{10} + \lambda_{-2}R_{x0} \tag{65}$$

$$\frac{dR_{20}}{dt} = 2k_{+2}LR_{00} - k_{-2}R_{20} - k_{+1}LR_{20} + k_{-1}R_{12} - k_{+2}LR_{20} + 2k_{-2}R_{22} - \lambda_{+1}R_{20} + \lambda_{-1}R_{x0} \tag{66}$$

$$\frac{dR_{11}}{dt} = k_{+1}LR_{10} - 2k_{-1}R_{11} - 2\lambda_{+2}R_{11} + \lambda_{-2}R_{x1} \tag{67}$$

$$\frac{dR_{12}}{dt} = k_{+2}LR_{10} - k_{-2}R_{12} + k_{+1}LR_{20} - k_{-1}R_{12} - \lambda_{+1}R_{12} + \lambda_{-1}R_{x1} - \lambda_{+2}R_{12} + \lambda_{-2}R_{x2} \tag{68}$$

$$\frac{dR_{22}}{dt} = k_{+2}LR_{20} - 2k_{-2}R_{22} - 2\lambda_{+1}R_{22} + \lambda_{-1}R_{x2} \tag{69}$$

$$\frac{dR_{x0}}{dt} = \lambda_{+2}R_{10} - \lambda_{-2}R_{x0} + \lambda_{+1}R_{20} - \lambda_{-1}R_{x0} - k_{+1}LR_{x1} + k_{-1}R_{x0} - k_{+2}LR_{x2} + k_{-2}R_{x0} \tag{70}$$

$$\frac{dR_{x1}}{dt} = 2\lambda_{+2}R_{11} - \lambda_{-2}R_{x1} + \lambda_{+1}R_{12} - \lambda_{-1}R_{x1} + k_{+1}LR_{x1} - k_{-1}R_{x0} \tag{71}$$

$$\frac{dR_{x2}}{dt} = 2\lambda_{+1}R_{22} - \lambda_{-1}R_{x2} + \lambda_{+2}R_{12} - \lambda_{-2}R_{x2} + k_{+2}LR_{x2} - k_{-2}R_{x0} \tag{72}$$

where the k and λ terms are rate constants, L is the concentration of free ligand, and the R variables are the concentrations of the receptor dimer species indicated in Figure F. From conservation of ligand and receptor, we also have the following algebraic equations:

$$L_T = L + R_{10} + R_{20} + R_{x0} + 2(R_{11} + R_{12} + R_{22} + R_{x1} + R_{x2}) \tag{73}$$

and

$$R_T = R_{00} + R_{10} + R_{20} + R_{11} + R_{12} + R_{22} + R_{x0} + R_{x1} + R_{x2} \tag{74}$$

where L_T is the total abundance of ligand and R_T is the total abundance of receptor dimer.

From inspection of Equations (64)–(74), we see that the form of these equations will be the same if we replace each concentration $X \in \{L, L_T, R_{00}, R_{10}, R_{20}, R_{11}, R_{12}, R_{22}, R_{x0}, R_{x1}, R_{x2}, R_T\}$

with $C \cdot X$ and, furthermore, we replace k_{+1} and k_{+2} with k_{+1}/C and k_{+2}/C, respectively, where C is a constant. Thus, ligand-receptor binding dynamics are unaffected by the scaling of Figure E, which we can now recognize as an example of the invariant scaling discussed immediately above with $C = 2$.

Listings of two BioNetGen input files are given in Figures G and H. The first file corresponds to the case where a receptor dimer is taken to have two copies of Y973 (Figure G). The second file corresponds to the case where a receptor dimer is taken to have only a single copy of Y973 (Figure H). The latter file is obtained from the former file through scaling. As can be easily confirmed, the corresponding simulation outputs are identical for the two models of Figures G and H, as would be expected. It should be noted that rescaling of concentrations is required for simulation outputs of the restructured model; examples of rescaling are included in the `functions` block of the BioNetGen input file shown in Figure H.

5 Closing remarks

The natural formulation of our IGF1R model implies a vast chemical reaction network, which is impracticable to derive. (Network generation is a computationally expensive procedure and large networks have large demands for allocation of computer memory.) Through decoupling, bunching, and scaling, we were able to transform the model into one that implies a much smaller network, making indirect simulation feasible. In other words, after restructuring, network generation followed by numerical integration of the corresponding ODEs becomes feasible. The network derived from the restructured model is smaller but not because information about system state has been lost. Rather, the rule-derived state variables (i.e., the concentrations of all potentially populated chemical species) are simply less redundant after restructuration. Each transformation (decoupling, bunching, and scaling) is elementary and straightforward. If these transformations are applied in different orders (or one is omitted), different reaction networks may/will be implied by the resulting model forms. Indeed, a given transformation may not result in making network generation less expensive. It is sufficient to find an adequate restructuration. There is no need to seek an optimum restructuration yielding a network of minimum size, because redundancy does little if any harm so long as network generation is feasible.

As noted earlier, we have presented restructuration as a body of model transformation techniques or strategies, because a modeler would ordinarily begin with a natural formulation of mechanistic knowledge. Natural model formulations have a tendency to imply large reaction networks. However, the truth of the matter is that the same essential model for a given system can be formulated *ab initio* in different ways and some of these are better than others. The restructuration strategies discussed here thus can be viewed as simply ideas about how to optimize model formulation for cases where one would like to apply an indirect simulation method in which a reaction network and the corresponding ODEs are first derived and then the ODEs are numerically integrated.

In this tutorial, we have assumed familiarity with the BioNetGen language (BNGL), the model-definition language of BioNetGen (Blinov et al., 2004; Harris et al., 2016). An informal description of BNGL is provided by Faeder et al. (2009). A formal description is provided by Hogg et al. (2014). Tutorials about the use of BNGL are available (Sekar and Faeder, 2012; Chylek et al., 2015). The utility of restructuration is not limited to users of BNGL. Restructuration is also relevant to users of related modeling languages, such as Kappa (Boutillier et al., 2018) and PySB (Lopez et al., 2013). We note that a tool is now available that enables automatic two-way translation between BNGL and Kappa (Suderman and Hlavacek, 2017) (https://github.com/RuleWorld/TRuML).

6 References

1. Birtwistle MR (2015) Analytical reduction of combinatorial complexity arising from multiple protein modification sites. *J R Soc Interface* **12**:20141215.

2. Blinov ML, Faeder JR, Goldstein B, Hlavacek WS (2004) BioNetGen: software for rule-based modeling of signal transduction based on the interactions of molecular domains. *Bioinformatics* **20**:3289–3291.

3. Blinov ML, Faeder JR, Goldstein B, Hlavacek WS (2006) A network model of early events in epidermal growth factor receptor signaling that accounts for combinatorial complexity. *BioSystems* **83**:136–151.

4. Borisov NM, Markevich NI, Hoek JB, Kholodenko BN (2005) Signaling through receptors and scaffolds: independent interactions reduce combinatorial complexity. *Biophys J* **89**:951–966.

5. Borisov NM, Markevich NI, Hoek JB, Kholodenko BN (2006) Trading the micro-world of combinatorial complexity for the macro-world of protein interaction domains. *BioSystems* **83**:152–166.

6. Borisov NM, Chistopolsky AS, Faeder JR, Kholodenko BN (2008) Domain-oriented reduction of rule-based network models. *IET Syst Biol* **2**:342–351.

7. Boutillier P, Ehrhard T, Krivine J (2017) Incremental update for graph rewriting. *Lect Notes Comput Sci* **10201**:201–228.

8. Boutillier P, Maasha M, Li X, Medina-Abarca HF, Krivine J, Feret J, Cristescu I, Forbes AG, Fontana W (2018) The Kappa platform for rule-based modeling. *Bioinformatics* **34**:i583–i592.

9. Bugenhagen SM, Beard DA (2012) Specification, construction, and exact reduction of state transition system models of biochemical processes. *J Chem Phys* **137**:154108.

10. Burgess AW, Cho HS, Eigenbrot C, Ferguson KM, Garrett TPJ, Leahy DJ, Lemmon MA, Sliwkowski MX, Ward CW, Yokoyama S (2003) An open-and-shut case? Recent insights into the activation of EGF/ErbB receptors. *Mol Cell* **12**:541–552.

11. Cardelli L, Tribastone M, Tschaikowski M, Vandin A (2017a) ERODE: a tool for the evaluation and reduction of ordinary differential equations. *Lect Notes Comput Sci* **10206**:310–328.

12. Cardelli L, Tribastone M, Tschaikowski M, Vandin A (2017b) Maximal aggregation of polynomial dynamical systems. *Proc Natl Acad Sci USA* **114**:10029–10034.

13. Chylek LA, Stites EC, Posner RG, Hlavacek WS (2013) Innovations of the rule-based modeling approach. In *Systems Biology: Integrative Biology and Simulation Tools*, Vol 1, Ch 9, pp 273–300 (Prokop A, Csukás B, Eds), Springer, Dordrecht.

14. Chylek LA, Harris LA, Tung CS, Faeder JR, Lopez CF, Hlavacek WS (2014) Rule-based modeling: a computational approach for studying biomolecular site dynamics in cell signaling systems. *Wiley Interdiscip Rev Syst Biol Med* **6**:13–36.

15. Chylek LA, Harris LA, Faeder JR, Hlavacek WS (2015) Modeling for (physical) biologists: an introduction to the rule-based approach. *Phys Biol* **12**:045007.

16. Colvin J Monine MI, Faeder JR, Hlavacek WS, Von Hoff DD, Posner RG (2009) Simulation of large-scale rule-based models. *Bioinformatics* **25**:910–917.

17. Colvin J Monine MI, Gutenkunst RN, Hlavacek WS, Von Hoff DD, Posner RG (2010) RuleMonkey: software for stochastic simulation of rule-based models. *BMC Bioinformatics* **11**:404.

18. Conzelmann H, Saez-Rodriguez J, Sauter T, Bullinger E, Allgöwer F, Gilles ED (2004) Reduction of mathematical models of signal transduction networks: simulation-based approach applied to EGF receptor signalling. *Sys Biol* **1**:159–169.

19. Conzelmann H, Fey D, Gilles ED (2008) Exact model reduction of combinatorial reaction networks. *BMC Syst Biol* **2**:78.

20. Danos V, Feret J, Fontana W, Krivine J (2007) Scalable simulation of cellular signaling networks. *Lect Notes Comput Sci* **4807**:139–157.

21. Faeder JR, Hlavacek WS, Reischl I, Blinov ML, Metzger H, Redondo A, Wofsy C, Goldstein B (2003) Investigation of early events in FcϵRI-mediated signaling using a detailed mathematical model. *J Immunol* **170**:3769–3781.

22. Faeder JR, Blinov ML, Goldstein B, Hlavacek WS (2005) Combinatorial complexity and dynamical restriction of network flows in signal transduction. *Syst Biol* **2**:5–15.

23. Faeder JR, Blinov ML, Hlavacek WS (2009) Rule-based modeling of biochemical systems with BioNetGen. *Methods Mol Biol* **500**:113–167.

24. Feret J, Danos V, Krivine J, Harmer R, Fontana W (2009) Internal coarse-graining of molecular systems. *Proc Natl Acad Sci USA* **106**:6453–6458.

25. Feret J, Henzinger T, Koeppl H, Petrov T (2012) Lumpability abstractions of rule-based systems. *Theor Comput Sci* **431**:137–164.

26. Feret J, Koeppl H, Petrov T (2013) Stochastic fragments: a framework for the exact reduction of the stochastic semantics of rule-based models. *Int J Softw Inform* **7**:527–604.

27. Gillespie DT (2007) Stochastic simulation of chemical kinetics. *Annu Rev Phys Chem* **58**:35–55.

28. Harris LA, Hogg JS, Tapia JJ, Sekar JA, Gupta S, Korsunsky I, Arora A, Barua D, Sheehan RP, Faeder JR (2016) BioNetGen 2.2: advances in rule-based modeling. *Bioinformatics* **32**:3366–3368.

29. Hlavacek WS, Faeder JR, Blinov ML, Perelson AS, Goldstein B (2003) The complexity of complexes in signal transduction. *Biotechnol Bioeng* **84**:783–794.

30. Hogg JS, Harris LA, Stover LJ, Nair NS, Faeder JR (2014) Exact hybrid particle/population simulation of rule-based models of biochemical systems. *PLOS Comput Biol* **10**:e1003544.

31. Kholodenko BN, Demin OV, Moehren G, Hoek JB (1999) Quantification of short term signaling by the epidermal growth factor receptor. *J Biol Chem* **274**:30169–30181.

32. Klinke DJ II, Finley SD (2012) Timescale analysis of rule-based biochemical reaction networks. *Biotechnol Prog* **28**:33–44.

33. Koschorrek M, Conzelmann H, Ebert S, Ederer M, Gilles ED (2007) Reduced modeling of signal transduction—a modular approach. *BMC Bioinformatics* **8**:336.

34. Lin YT, Chylek LA, Lemons, NW, Hlavacek WS (2018) Using equation-free computation to accelerate network-free stochastic simulation of chemical kinetics. *J Phys Chem B* **122**:6351–6356.

35. Lopez CF, Muhlich JL, Bachman JA, Sorger PK (2013) Programming biological models in Python using PySB. *Mol Syst Biol* **9**:646.

36. Petrov T, Feret J, Koeppl H (2012) Reconstructing species-based dynamics from reduced stochastic rule-based models. In *Proceedings of the 2012 Winter Simulation Conference* (Laroque C, Himmelspach J, Pasupathy R, Rose O, Uhrmacher AM, Eds), IEEE, Los Alamitos.

37. Sekar JA, Faeder JR (2012) Rule-based modeling of signal transduction: a primer. *Methods Mol Biol* **880**:139–218.

38. Sneddon MW, Faeder JR, Emonet T (2011) Efficient modeling, simulation and coarse-graining of biological complexity with NFsim. *Nat Methods* **8**:177–183.

39. Sorkin A, Helin K, Waters CM, Carpenter G, Beguinot L (1992) Multiple autophosphorylation sites of the epidermal growth factor receptor are essential for receptor kinase activity and internalization. Contrasting significance of tyrosine 992 in the native and truncated receptors. *J Biol Chem* **267**:8672–8678.

40. Stefan MI, Bartol TM, Sejnowski TJ, Kennedy MB (2014) Multi-state modeling of biomolecules. *PLOS Comput Biol* **10**:e1003844.

41. Suderman R, Hlavacek WS (2017) TRuML: a translator for rule-based modeling languages. In *Proceedings of the 8th ACM International Conference on Bioinformatics, Computational Biology, and Health Informatics* (ACM-BCB '17, Boston, MA, August 20-23, 2017), pp 372-377. ACM Press, New York.

42. Suderman R, Mitra ED, Lin YT, Erickson KE, Feng S, Hlavacek WS (2018) Generalizing Gillespie's direct method to enable network-free simulations. *Bull Math Biol* doi: 10.1007/s11538-018-0418-2

43. Yang J, Monine MI, Faeder JR, Hlavacek WS (2008) Kinetic Monte Carlo method for rule-based modeling of biochemical networks. *Phys Rev E* **78**:031910.

44. Yang J, Hlavacek WS (2011) The efficiency of reactant site sampling in network-free simulation of rule-based models for biochemical systems. *Phys Biol* **8**:055009.

```
begin model
  begin parameters
    kpX 1
    kmX 1
    kpY 1
    kmY 1
    kpZ 1
    kmZ 1
    RTOT 100
  end parameters
  begin molecule types
    R(X~0~1,Y~0~1,Z~0~1)
  end molecule types
  begin seed species
    R(X~0,Y~0,Z~0) RTOT
  end seed species
  begin observables
    Species R111 R(X~1,Y~1,Z~1)
  end observables
  begin reaction rules
    R(X~0)<->R(X~1) kpX,kmX
    R(Y~0)<->R(Y~1) kpY,kmY
    R(Z~0)<->R(Z~1) kpZ,kmZ
  end reaction rules
end model
begin actions
  generate_network({overwrite=>1})
  simulate({method=>"ode",t_end=>10,n_steps=>100})
end actions
```

Figure A: This BioNetGen input file listing shows the definition of a naturally formulated rule-based model wherein a receptor is represented as having three sites that can each toggle spontaneously between two different internal states, 0 and 1. The model is defined using the BioNetGen language (BNGL) (Faeder et al., 2009; Hogg et al., 2014). The observable `R111` is defined as a simulation output; this quantity reports the time-dependent concentration of receptor with all three sites in the "1" state. This version of the model implies a reaction network consisting of 8 chemical species and 24 reactions. An electronic copy of the BioNetGen input file displayed here (`before_decoupling.bngl`) is available online at RuleHub (https://github.com/RuleWorld/RuleHub).

```
begin model
  begin parameters
    kpX 1
    kmX 1
    kpY 1
    kmY 1
    kpZ 1
    kmZ 1
    RTOT 100
  end parameters
  begin molecule types
    X(S~0~1)
    Y(S~0~1)
    Z(S~0~1)
  end molecule types
  begin seed species
    X(S~0) RTOT
    Y(S~0) RTOT
    Z(S~0) RTOT
  end seed species
  begin observables
    Species X1 X(S~1)
    Species Y1 Y(S~1)
    Species Z1 Z(S~1)
  end observables
  begin functions
    R111()=(X1/RTOT)*(Y1/RTOT)*(Z1/RTOT)*RTOT
  end functions
  begin reaction rules
    X(S~0)<->X(S~1) kpX,kmX
    Y(S~0)<->Y(S~1) kpY,kmY
    Z(S~0)<->Z(S~1) kpZ,kmZ
  end reaction rules
end model
begin actions
  generate_network({overwrite=>1})
  simulate({method=>"ode",t_end=>10,n_steps=>100,\
            print_functions=>1})
end actions
```

Figure B: This BioNetGen input file listing shows a restructured formulation of the model of Figure A. In this formulation of the model, the three receptor sites are each represented independently. The global function `R111()` is defined as a simulation output; this quantity reports the time-dependent concentration of receptor with all three sites in the “1” state. As can be easily confirmed, the `R111()` time course generated when BioNetGen processes this file is indistinguishable from the `R111` time course generated when BioNetGen processes the file of Figure A. This version of the model implies a reaction network consisting of 6 chemical species and 6 reactions. An electronic copy of the BioNetGen input file displayed here (`after_decoupling.bngl`) is available online at RuleHub (https://github.com/RuleWorld/RuleHub).

```
begin model
  begin parameters
    NA 6.02214076e23 # molecules per mol
    Vecf 1e-9 # L per cell
    EGFtot 25e-9*(NA*Vecf) # copies per cell
    EGFRtot 1e5 # copies per cell
    KD 1e-9 # M
    kr 5e-3 # /s
    kf=(kr/KD)/(NA*Vecf) # /(molecule/cell)/s
    kpx 3e-5 # /(molecule/cell)/s
    kmx 0.3 # /s
    kp 0.1 # /s
    kdp 0.1 # /s
  end parameters
  begin molecule types
    EGF(EGFL)
    EGFR(I_III,II,Y1016~0~P,Y1092~0~P,Y1110~0~P,Y1172~0~P,Y1197~0~P)
  end molecule types
  begin seed species
    EGF(EGFL) EGFtot
    EGFR(I_III,II,Y1016~0,Y1092~0,Y1110~0,Y1172~0,Y1197~0) EGFRtot
  end seed species
  begin observables
    Molecules EGFR_monomers EGFR(II)
    Molecules EGFR_in_dimers EGFR(II!+)
    Molecules pY1092 EGFR(Y1092~P!?)
  end observables
  begin reaction rules
    EGF(EGFL)+EGFR(I_III,II)<->EGF(EGFL!1).EGFR(I_III!1,II) kf,kr
    EGFR(I_III!+,II)+EGFR(I_III!+,II)<->EGFR(I_III!+,II!1).EGFR(I_III!+,II!1) kpx,kmx
    EGFR(II!+,Y1016~0)->EGFR(II!+,Y1016~P) kp
    EGFR(II!+,Y1092~0)->EGFR(II!+,Y1092~P) kp
    EGFR(II!+,Y1110~0)->EGFR(II!+,Y1110~P) kp
    EGFR(II!+,Y1172~0)->EGFR(II!+,Y1172~P) kp
    EGFR(II!+,Y1197~0)->EGFR(II!+,Y1197~P) kp
    EGFR(Y1016~P)->EGFR(Y1016~0) kdp
    EGFR(Y1092~P)->EGFR(Y1092~0) kdp
    EGFR(Y1110~P)->EGFR(Y1110~0) kdp
    EGFR(Y1172~P)->EGFR(Y1172~0) kdp
    EGFR(Y1197~P)->EGFR(Y1197~0) kdp
  end reaction rules
end model
begin actions
  generate_network({overwrite=>1})
  simulate({method=>"ode",t_end=>60,n_steps=>120})
end actions
```

Figure C: A (simplified) model for EGF-induced dimerization of EGFR and dimerization-dependent EGFR autophosphorylation. The model accounts for five sites of autophosphorylation (Sorkin et al., 1992). We use UniProt numbering of amino acid residues. (One can subtract 24 to obtain the traditional numbering.) Dimers of EGFR are represented as connected pairs of EGFR monomers; see the right-hand side of the second rule, which provides a formal representation of reversible EGFR dimerization. An electronic copy of the BioNetGen input file displayed here (`before_bunching.bngl`) is available online at RuleHub (https://github.com/RuleWorld/RuleHub).

```
begin model
  begin parameters

    ⋮

  end parameters
  begin molecule types
    EGF(EGFL)
    EGFR(I_III,II~m~d,Y1016~0~P,Y1092~0~P,Y1110~0~P,Y1172~0~P,Y1197~0~P)
  end molecule types
  begin seed species
    EGF(EGFL) EGFtot
    EGFR(I_III,II~m,Y1016~0,Y1092~0,Y1110~0,Y1172~0,Y1197~0) EGFRtot
  end seed species
  begin observables
    Molecules EGFR_monomers EGFR(II~m)
    Molecules EGFR_in_dimers EGFR(II~d)
    Molecules pY1092 EGFR(Y1092~P!?)
  end observables
  begin reaction rules
    EGF(EGFL)+EGFR(I_III,II~m)<->EGF(EGFL!1).EGFR(I_III!1,II~m) kf,kr
    EGFR(I_III!+,II~m)+EGFR(I_III!+,II~m)->EGFR(I_III!+,II~d)+EGFR(I_III!+,II~d) kpx
    EGFR(I_III!+,II~d)->EGFR(I_III!+,II~m) kmx
    EGFR(II~d,Y1016~0)->EGFR(II~d,Y1016~P) kp
    EGFR(II~d,Y1092~0)->EGFR(II~d,Y1092~P) kp
    EGFR(II~d,Y1110~0)->EGFR(II~d,Y1110~P) kp
    EGFR(II~d,Y1172~0)->EGFR(II~d,Y1172~P) kp
    EGFR(II~d,Y1197~0)->EGFR(II~d,Y1197~P) kp
    EGFR(Y1016~P)->EGFR(Y1016~0) kdp
    EGFR(Y1092~P)->EGFR(Y1092~0) kdp
    EGFR(Y1110~P)->EGFR(Y1110~0) kdp
    EGFR(Y1172~P)->EGFR(Y1172~0) kdp
    EGFR(Y1197~P)->EGFR(Y1197~0) kdp
  end reaction rules
end model
begin actions
  generate_network({overwrite=>1})
  simulate({method=>"ode",t_end=>60,n_steps=>120})
end actions
```

Figure D: A restructuring of the model of Figure C. In this formulation of the model, the `EGFR` molecule type definition bunches together the representation of both receptor monomers and dimers, whereas in the original model, receptor dimers are represented as an assembly of receptor monomers (i.e., dimers are represented by connectivity). The bunching is accomplished by introducing two internal states for site `II` in `EGFR`: `m` (to indicate a monomeric receptor) and `d` (to indicate a receptor in a dimer). Note that the rule in the original model for reversible dimerization has been replaced by two unidirectional rules, one for dimerization and one for dimer dissociation. As can be easily confirmed, the simulation outputs generated when BioNetGen processes this input file are indistinguishable from those generated when BioNetGen processes the input file shown in Figure C. Here, the `generate_network` command produces a reaction network consisting of 97 chemical species and 944 reactions. In contrast, for the original model formulation of Figure C, the `generate_network` command produces a reaction network consisting of 593 chemical species and 6,400 reactions. The content of the parameters block is the same as in Figure C. An electronic copy of the BioNetGen input file displayed here (`after_bunching.bngl`) is available online at RuleHub (https://github.com/RuleWorld/RuleHub).

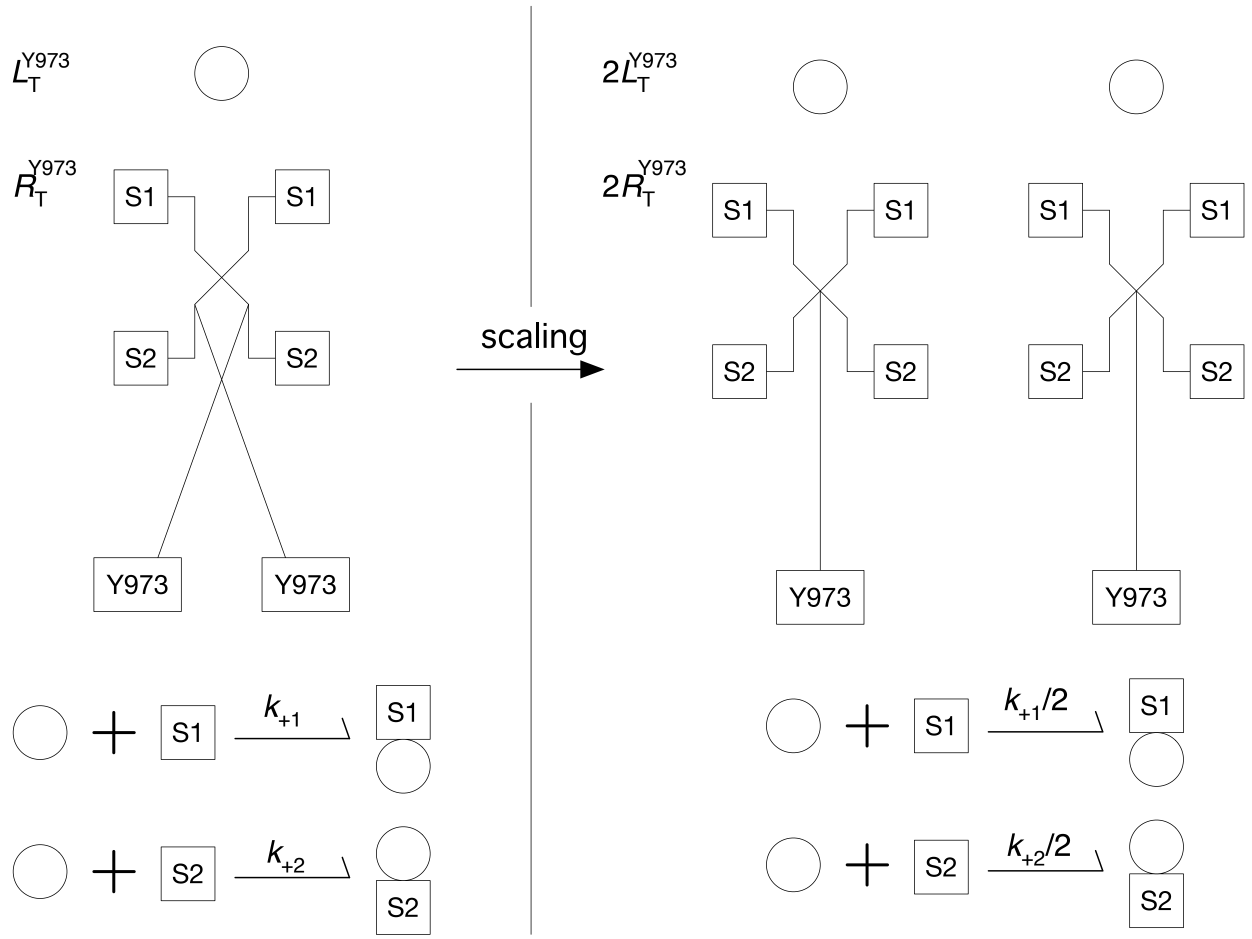

Figure E: An example of a scaling transformation. Here, a receptor dimer (dimeric IGF1R) with two identical copies of a tyrosine (Y973) is represented naturally at left. After scaling, the receptor dimer is represented as having only a single copy of the tyrosine. To compensate for the elimination of site degeneracy, model parameters are scaled. The total ligand and receptor dimer abundances are each increased by a factor of 2, and the rate constants that characterize ligand (IGF1) binding to IGF1R, k_{+1} and k_{+2}, are each decreased by a factor of 2.

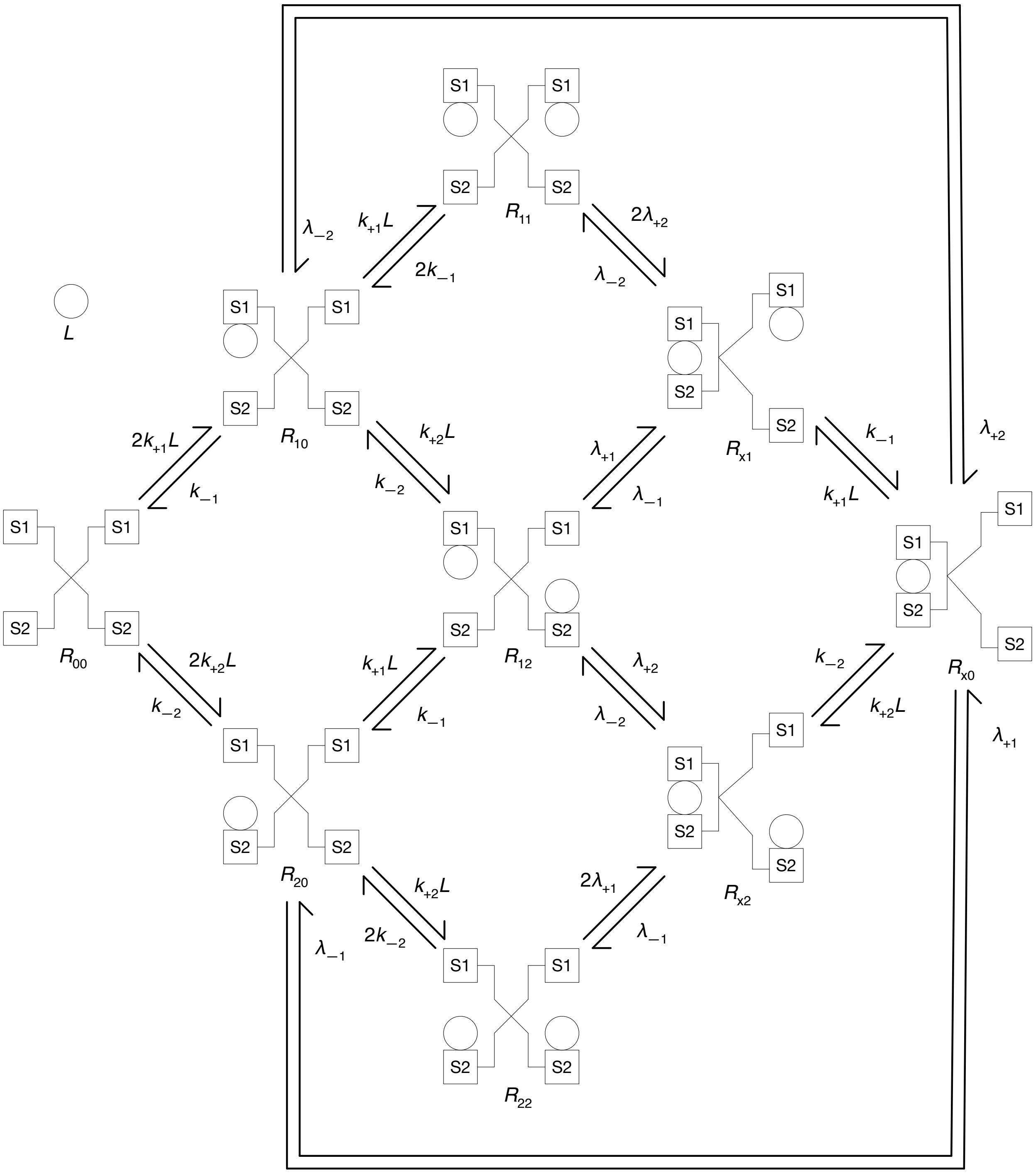

Figure F: Reaction scheme for IGF1 interaction with dimeric IGF1R. See the text for discussion.

```
begin model
  begin parameters
    NA 6.02214076e23
    V 1e-9
    LT 1e-9*(NA*V)
    RT 1e5
    kp1 2.8e5/(NA*V)
    km1 5e-2
    kp2 1.5e4/(NA*V)
    km2 1.9e-4
    lp1 5.6e-3
    lm1 1.9e-5
    lp2 5.2e1
    lm2 1.3e-2
    kp 0.5
    kdp 0.1
  end parameters
  begin molecule types
    L()
    R(P~0~1~2~x,P~0~1~2~x,Y~0~P,Y~0~P)
  end molecule types
  begin seed species
    L() LT
    R(P~0,P~0,Y~0,Y~0) RT
  end seed species
  begin observables
    Species freeL L()
    Molecules boundL R(P~1) R(P~2) R(P~x)
    Species freeR R(P~0,P~0)
    Molecules Crosslink R(P~x)
    Molecules pY R(Y~P!?)
  end observables
  begin reaction rules
    R(P~0)+L()<->R(P~1) kp1,km1
    R(P~0)+L()<->R(P~2) kp2,km2
    %x:R(P~1)<->R(P~x) if(Crosslink(x)>0,0,lp2),lm2
    %x:R(P~2)<->R(P~x) if(Crosslink(x)>0,0,lp1),lm1
    R(P~x,Y~0)->R(P~x,Y~P) kp
    R(Y~P)->R(Y~0) kdp
  end reaction rules
end model
begin actions
  generate_network({overwrite=>1})
  simulate({method=>"ode",t_end=>12000,n_steps=>120})
end actions
```

Figure G: A simple rule-based model for IGF1-IGF1R interaction and IGF1-stimulated autophosphorylation of IGF1R. This model is consistent with the reaction scheme of Figure F. Each receptor dimer `R` is taken to have two identical copies of a ligand binding pocket `P` and two identical copies of a tyrosine residue `Y`. An electronic copy of the BioNetGen input file displayed here (`before_scaling.bngl`) is available online at RuleHub (https://github.com/RuleWorld/RuleHub). A file that provides a direct encoding of the reaction scheme of Figure F (`check_scaling.bngl`) is also available at RuleHub. For a different, more natural formulation of this model, see Chylek et al. (2014).

```
begin model
  begin parameters
    NA 6.02214076e23
    V 1e-9
    LT 2*1e-9*(NA*V)
    RT 2*1e5
    kp1 0.5*2.8e5/(NA*V)
    km1 5e-2
    kp2 0.5*1.5e4/(NA*V)
    km2 1.9e-4
    lp1 5.6e-3
    lm1 1.9e-5
    lp2 5.2e1
    lm2 1.3e-2
    kp 0.5
    kdp 0.1
  end parameters
  begin molecule types
    L()
    R(P~0~1~2~x,P~0~1~2~x,Y~0~P)
  end molecule types
  begin seed species
    L() LT
    R(P~0,P~0,Y~0) RT
  end seed species
  begin observables
    Species freeL L()
    Molecules boundL R(P~1) R(P~2) R(P~x)
    Species freeR R(P~0,P~0)
    Molecules Crosslink R(P~x)
    Molecules pY R(Y~P!?)
  end observables
  begin functions
    rescaled_freeL()=freeL/2
    rescaled_boundL()=boundL/2
    rescaled_freeR()=freeR/2
    rescaled_Crosslink()=Crosslink/2
  end functions
  begin reaction rules
    R(P~0)+L()<->R(P~1) kp1,km1
    R(P~0)+L()<->R(P~2) kp2,km2
    %x:R(P~1)<->R(P~x) if(Crosslink(x)>0,0,lp2),lm2
    %x:R(P~2)<->R(P~x) if(Crosslink(x)>0,0,lp1),lm1
    R(P~x,Y~0)->R(P~x,Y~P) kp
    R(Y~P)->R(Y~0) kdp
  end reaction rules
end model
begin actions
  generate_network({overwrite=>1})
  simulate({method=>"ode",t_end=>12000,n_steps=>120,\
           print_functions=>1})
end actions
```

Figure H: A restructured formulation of the model of Figure G. The original model has been modified through scaling. In the restructured model, each receptor dimer `R` is represented as having a single tyrosine site `Y` (vs. two identical copies of this site as in the original model). Note that the parameters `LT`, `RT`, `kp1`, and `kp2` have each been scaled to compensate for the elimination of `Y` site degeneracy. Here, the `generate_network` command produces a reaction network consisting of 21 chemical species and 78 reactions. In contrast, for the original model formulation of Figure G, the `generate_network` command produces a reaction network consisting of 31 chemical species and 124 reactions. An electronic copy of the BioNetGen input file displayed here (`after_scaling.bngl`) is available online at RuleHub (https://github.com/RuleWorld/RuleHub).
