# Supplementary material for: Modeling cell line-specific recruitment of signaling proteins to the insulin-like growth factor 1 receptor
Source: PLoS Comput Biol. 2019 Jan 17;15(1):e1006706. doi: 10.1371/journal.pcbi.1006706 (PMC6353226; doi:10.1371/journal.pcbi.1006706)
Supplement: S3 File — We derive analytical equations to predict binding of signaling proteins to an RTK and compare results to those of the numerical simulation described in the main text. Furthermore, we present a simplified set of equations, which provides a rule of thumb for estimating rank. (PDF) [file pcbi.1006706.s003.pdf]

S3 File

Derivation of the analytical formulation for predicting equilibrium binding of SH2 and PTB binding partners to an RTK

Receptor tyrosine kinases (RTKs) have many binding sites, and a binding partner (X_i, i = 1, …, N) can bind to some of these sites with K_D values denoted K_{ij}, j = 1, …, M_j. Our main assumption is that only one partner can bind to an RTK at a time, because of steric restrictions. Thus, each binding site on an RTK competes for binding partners, and for each partner X_i (i = 1, …, N) there is a set K_{ij} (j = 1, …, M_j) of K_D values that describe the binding of X_i to site j. Because usually a partner X_i cannot bind to all RTK sites, the number M_j indicates the number of sites to which X_i binds.

Our main assumption about fully competitive binding allows us to introduce RTK fractions (R_{ij}) that correspond to a complex (R_{ij}) of partner X_i bound to active RTK at site j at steady state:

$$R_{ij}=R_j X_i=\frac{R*X_i}{K_{ij}} \qquad \text{(S1)}$$

Here X_i and R are the concentrations of free partner and free active RTK, R_j is the concentration of free site j, and K_{ij} is the K_D of X_i binding to site j on the RTK.

We denote by R^{Tot} the total active RTK concentration and by X_i^{Tot} the total partner X_i abundance. The total active RTK concentration (R^{Tot}) depends on time, but we consider a steady state. The following are the moiety conservation equations (i.e., balance equations):

$$X_i+\sum_{j=1}^{Mi}\frac{R*X_i}{K_{ij}}=X_i^{Tot} \qquad \text{(S2)}$$

$$R+\sum_{i=1}^{N}\sum_{j=1}^{Mi}\frac{R*X_i}{K_{ij}}=R^{Tot} \qquad \text{(S3)}$$

The affinity of partner X_i for binding site j equals $1/K_{ij}$. For protein X_i, we designate the sum of its affinities for RTK sites to which X_i binds by $1/K_i$:

$$\sum_{j=1}^{Mi}\frac{1}{K_{ij}}=\frac{1}{K_i} \qquad \text{(S4)}$$

The quantity K_i can be termed the effective K_D for partner X_i (it is a reciprocal of the sum of the affinities of partner X_i for binding sites j on RTK). Then, we rewrite Eq. S2 as follows:

$$X_i+\frac{R*X_i}{K_i}=X_i^{Tot} \qquad \text{(S5)}$$

From Eq. S5, we can express X_i in terms of R, X_i^{Tot} and K_i as:

$$X_i = \frac{X_i^{Tot}}{1 + R/K_i} \quad \text{(S6)}$$

Substituting Eq. S6 into Eq. S3, we readily obtain the free active RTK concentration, R, from the following equation:

$$R\left(1 + \sum_{i=1}^{N} \frac{X_i^{Tot}}{K_i + R}\right) = R^{Tot} \quad \text{(S7)}$$

Each free concentration X_i can be obtained by substituting the solution R of Eq. S7 into Eq. S6. Each RTK fraction R_{ij} which corresponds to a complex of RTK and the partner X_i that has bound the RTK site j can be obtained by substituting the solution R of Eq. S7 and Eqs. S6 and S1:

$$R_j X_i = \frac{R * X_i^{Tot}}{\left(1 + R/K_i\right) K_{ij}} \quad \text{(S8)}$$

Thus, the total bound fraction of partner X_i is the sum of $R_j X_i$ over all binding sites:

$$X_i^{Bound} = \sum_{j=1}^{Mi} R_j X_i = \frac{R * X_i^{Tot}}{\left(1 + R/K_i\right)} \sum_{j=1}^{Mi} \frac{1}{K_{ij}} = \frac{R * X_i^{Tot}}{K_i + R} \quad \text{(S9)}$$

We used the analytical equilibrium binding model (Eqs. S7 and S9, solved in Mathematica) to estimate the concentrations of the bound forms of RTK partners upon IGF1 stimulation. The rank order predictions from the numerical simulations of the full model coincide well with those obtained from the simplified analytical model (Eq. S7 and S9) when R^{Tot} = 40 nM, with only six inconsistencies in rank order (see Table 2 in the main text, and Table A below). The inconsistencies likely arise from the analytical model assumption that only one binding partner can occupy an active RTK at a time, whereas the numerical model accounts for the dynamics of phosphorylation and binding events, making possible simultaneous binding of multiple partners. This finding is consistent with wider comparison of results from the analytical model and numerical simulations for each of the cell line-specific models. Overall, the rank order predictions from analytical model are more consistent with those from numerical calculations if the concentration of RTK free from binding partners is higher, thus improving the likliehood that each RTK molecule is bound only to one partner.

Table A. Comparison of rank ordering of RTK binding partners in HeLa S3 cells obtained from numerical calculations and simplified analytical model with R^{Tot} = 40 nM.

Protein	Rank obtained from numerical simulations of the full model	Rank obtained from simplified analytical model (Equations S7 and S9)
SHC1	1	6
CRKL	2	2
ABL2	3	3
STAT1	4	1
VAV2	5	4
YES1	6	5

RASA1	7	7
PI3KR2	8	8
PLCG2	9	10
PI3KR3	10	9
SRC	11	11
NCK2	12	12
PI3KR1	13	13
SYK	14	14
BLK	15	15
IRS1	16	16

In summary, the analytical model correctly predicts the overall structure of rank order of binding partners and may serve as a useful tool for studying the processes of recruitment of signaling proteins to RTKs.

A simplified solution to the analytical equilibrium binding model

We subdivided all RTK binding partners X_i into two groups according to their effective K_D values (Eq. S4 and denoted by K_i):

1. Those with K_i larger than R^{tot} ($K_i \geq R^{tot}$)
2. Those with K_i smaller than R^{Tot} ($K_i < R^{tot}$)

If $K_i \geq R^{tot}$, Eq. S9 can be approximated as a linear function of the free active RTK concentration:

$$X_i^{Bound} \approx \frac{R * X_i^{Tot}}{K_i} \qquad \text{(S10)}$$

Eq. S10 means that the bound fractions of partners with large effective K_D values (K_i) are proportional to their total abundances divided by K_i, potentially justifying the metric X_i^{Tot}/K_i, but only for those proteins that bind to RTK with $K_i \geq R^{tot}$. Accordingly, the concentration of free such proteins are approximated as:

$$X_i \approx X_i^{Tot}\left(1 - R/K_i\right) \qquad \text{(S11)}$$

Eq. S11 corresponds to the linear term of the Taylor expansion of Eq. S6. Using the approximatation in Eq. S10, the balance Eq. S7 can be written as follows, where Ki is large if $K_i \geq R^{tot}$ and small if $K_i < R^{tot}$:

$$R\left(1 + \sum_{i,\,Large\,Ki} \frac{X_i^{Tot}}{K_i} + \sum_{i,\,Small\,Ki} \frac{X_i^{Tot}}{K_i + R}\right) \approx R^{Tot} \qquad \text{(S12)}$$

If $K_i < R^{tot}$, and we consider exceptionally tightly-binding partners with K_i in the small nanomolar range, we can conjecture from Eq. S12 that the appropriate metric for predicting rank order will be their total abundances, X_i^{Tot}. The bound fractions of proteins that bind with K_i less than R^{tot} but greater than very small K_i can be found by solving Eq. S12. Previous numerical calculations show that for these proteins, the rank order of binding mainly depends on the total abundances X_i^{Tot}, although it also slightly depends on K_i and R^{tot}.

To summarize, a simplified, “rule of thumb” approach to ranking binding partners can be obtained as follows:

- For $K_i \geq R^{Tot}$, rank according to X_i^{Tot} / K_i
- For $K_i < R^{Tot}$, rank according to Eq. S10-S12
- If $K_i < R^{Tot}$ and K_i is very small (~ 10^{-9} M), rank according to X_i^{Tot}.

Concentrations of bound partners X_i^{Bound} calculated from Eqs. S10-S12 differ no more than 15% from solutions of Eq. S7 and S9.
