# Supplementary figures and images for: Modeling cell line-specific recruitment of signaling proteins to the insulin-like growth factor 1 receptor
Source: PLoS Comput Biol. 2019 Jan 17;15(1):e1006706. doi: 10.1371/journal.pcbi.1006706 (PMC6353226; doi:10.1371/journal.pcbi.1006706)

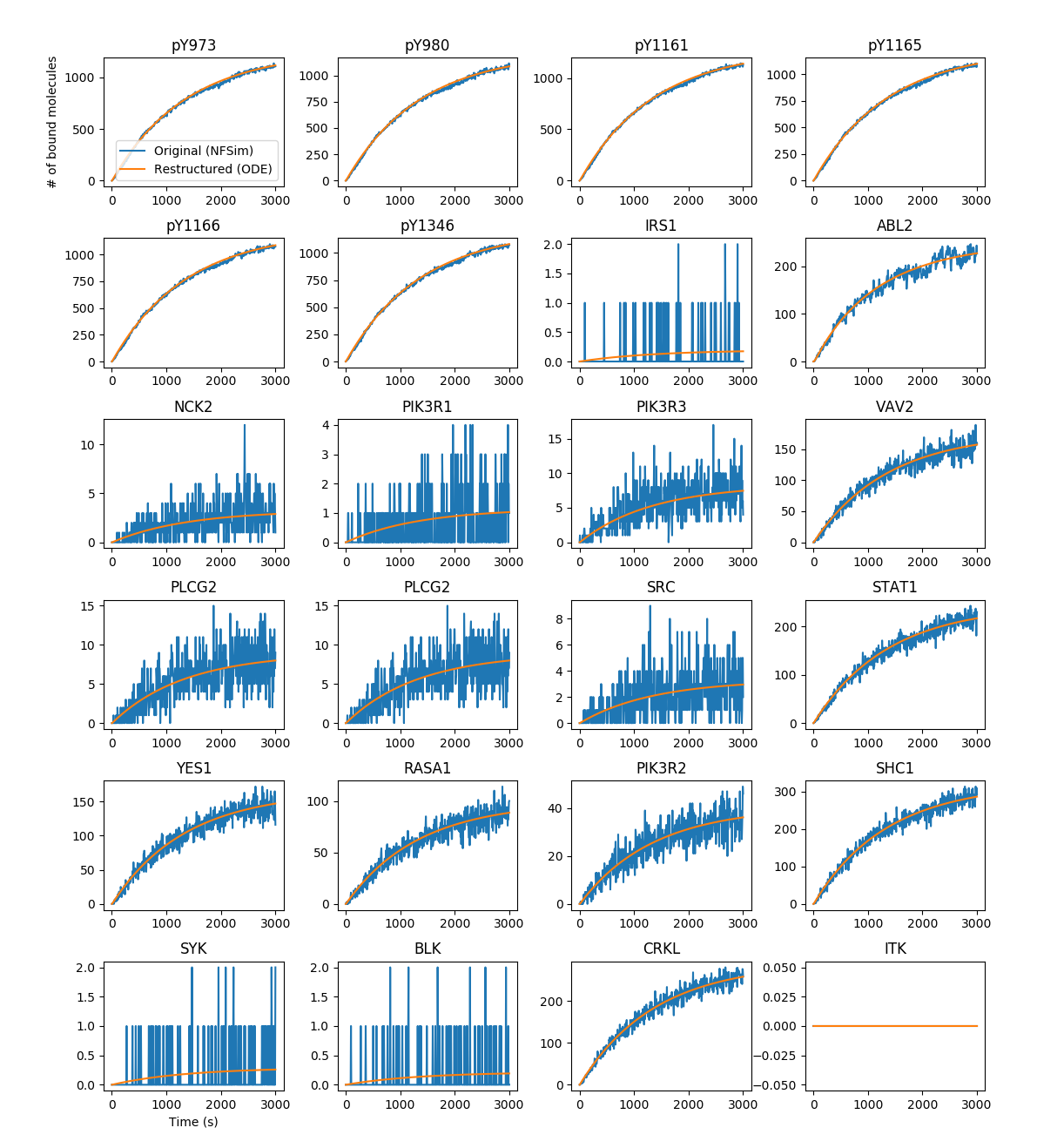

Supplement: S1 Fig — Plots show time courses of bound phosphotyrosine sites and bound signaling proteins from simulations of the HeLa S3 model in the natural formulation and the restructured formulation. (TIFF) [file pcbi.1006706.s007.tiff]

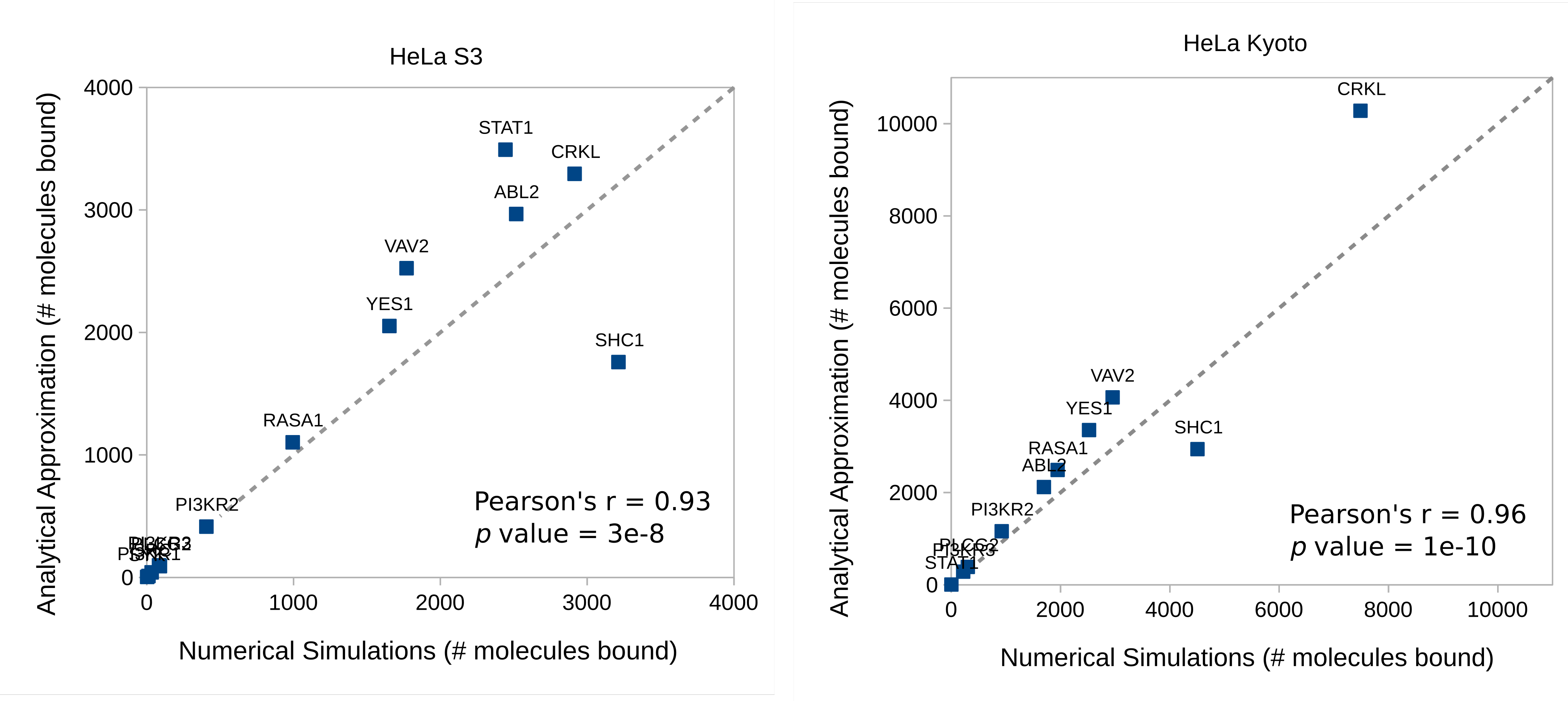

Supplement: S3 Fig — Plots show the number of molecules of each protein bound at steady state predicted by either numerical simulations (x-axis) or the analytical approximation (y-axis). A dashed gray line on the diagonal illustrates perfect agreement. The Pearson’s correlation coefficient and p value are displayed for each dataset (calculated using R software’s cor.test). (TIFF) [file pcbi.1006706.s009.tiff]

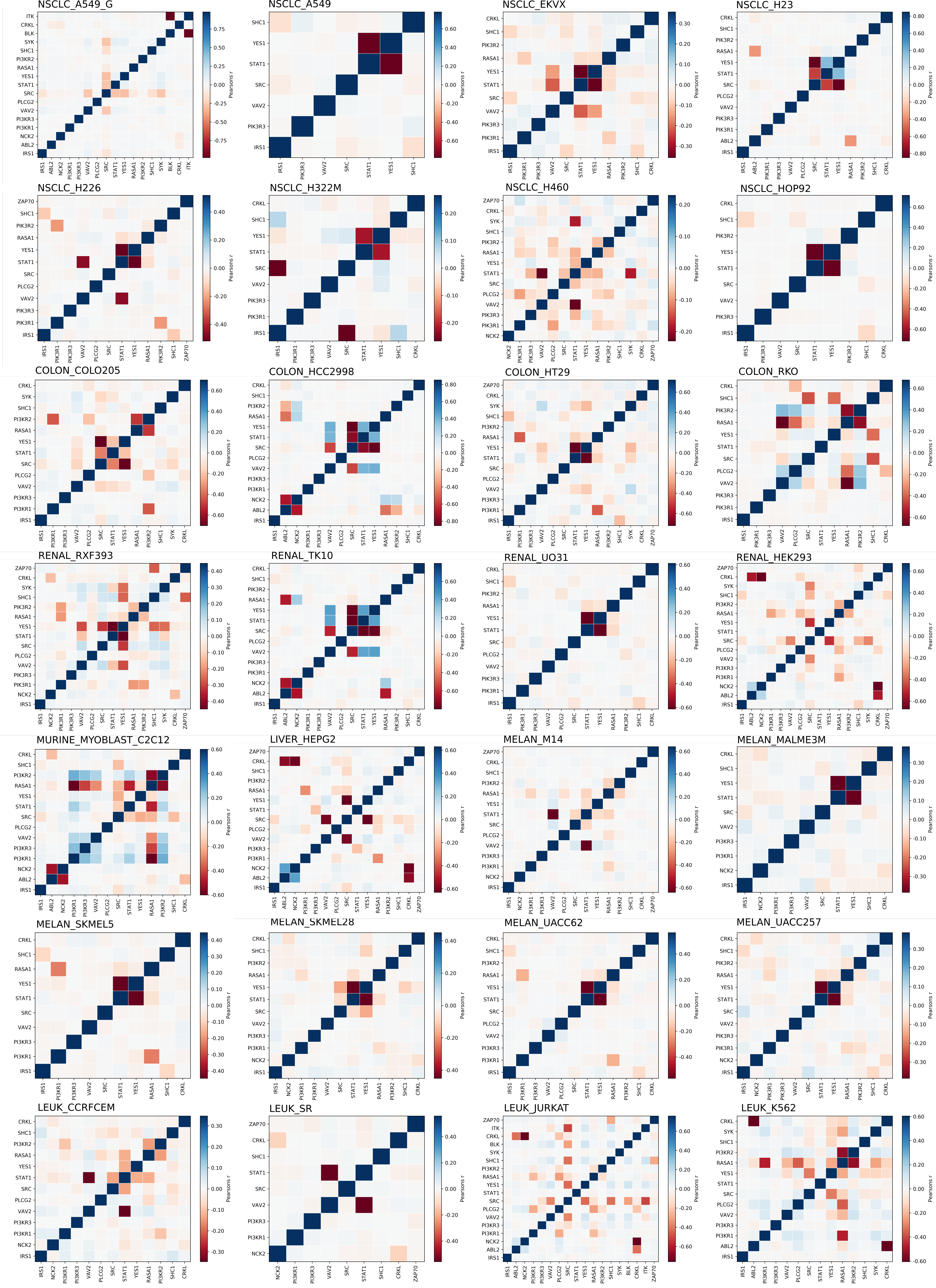

Supplement: S4 Fig — Red indicates a negative Pearson’s r, blue indicates a positive Pearson’s r, and white indicates no correlation between each pair of proteins. (TIFF) [file pcbi.1006706.s010.tiff]

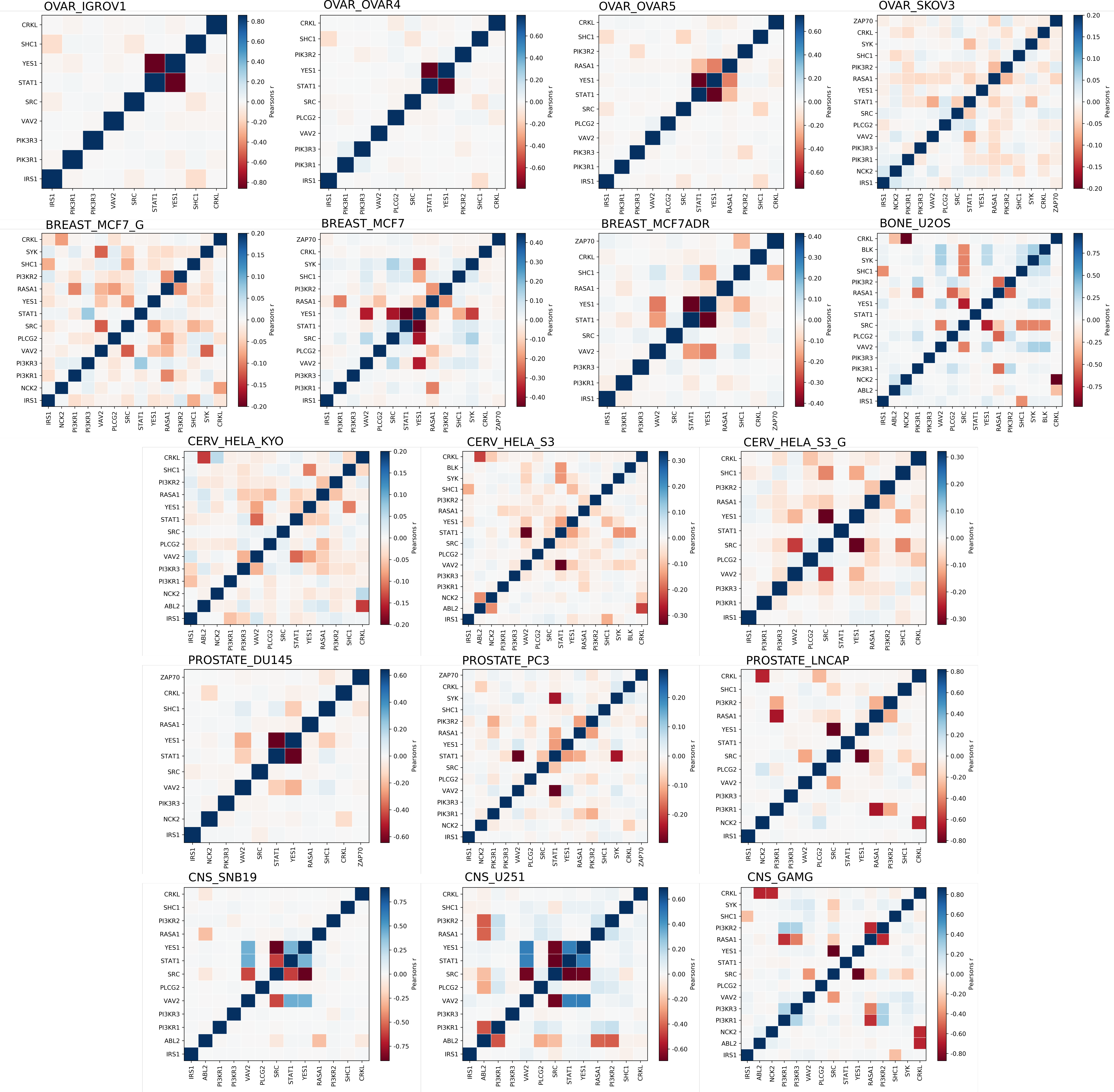

Supplement: S5 Fig — Red indicates a negative Pearson’s r, blue indicates a positive Pearson’s r, and white indicates no correlation between each pair of proteins. (TIFF) [file pcbi.1006706.s011.tiff]
