# Supplementary material for: Modeling cell line-specific recruitment of signaling proteins to the insulin-like growth factor 1 receptor
Source: PLoS Comput Biol. 2019 Jan 17;15(1):e1006706. doi: 10.1371/journal.pcbi.1006706 (PMC6353226; doi:10.1371/journal.pcbi.1006706)
Supplement: S2 Fig — Cartoons of (A) bunching (B) decoupling, and (C-D) scaling are shown. (A) We can couple an S1 site from one IGF1R monomer and the S2 site from the other IGF1R monomer into one binding pocket, P. In the natural formulation, four different binding sites can be either free or bound to IGF1. In the restructured formulation, two binding pockets can each be free (white circle), bound to IGF1 (gray circle with IGF1), or crosslinked (black circle with IGF1). (B) We decouple each of the phosphotyrosine sites from the others, since the state of one site does not influence the state of any other site. In the restructured formulation, we consider six forms of the receptor, each with only one possible tyrosine residue. (C) Each phosphotyrosine residue can be either dephosphorylated, phosphorylated and free (green circle), or phosphorylated and bound (green circle plus yellow pentagon). If we consider receptor monomers instead of dimers, the minimum number of possible states is reduced from six to three. (D) Upon the above restructuring, to preserve mass-action kinetics, the rate constant for ligand binding must be halved and the total receptor and ligand concentrations must be doubled. (PDF) [file pcbi.1006706.s008.pdf]

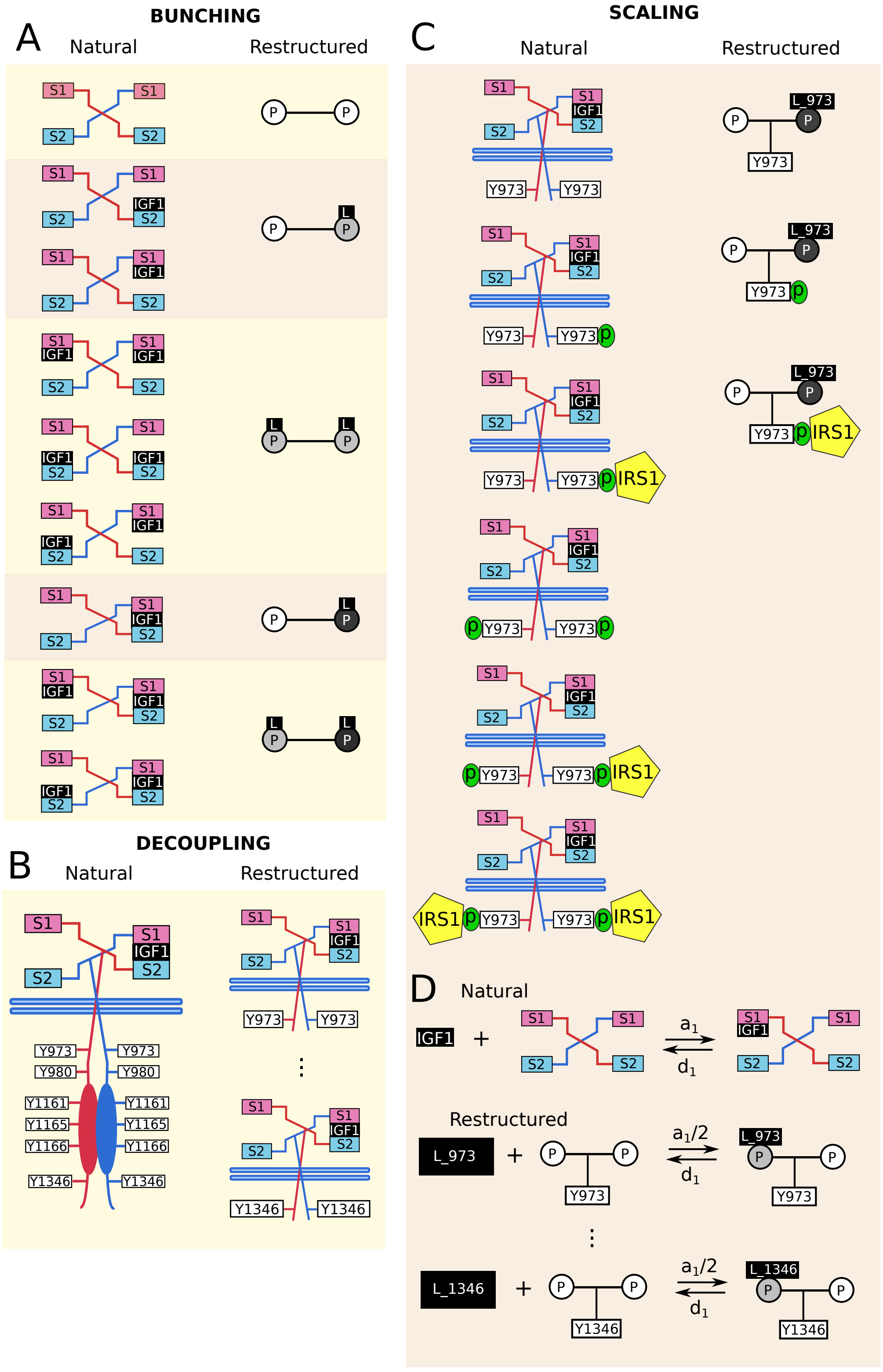
BUNCHING
A
Natural
Restructured
S1
S2
IGF1
P
L
SCALING
C
Natural
Restructured
L_973
Y973
p
IRS1
DECOUPLING
B
Natural
Restructured
Y973
Y980
Y1161
Y1165
Y1166
Y1346
D
Natural
IGF1
+
a_1
d_1
Restructured
L_973
$a_1/2$
L_1346
Y1346
